# Supplementary material for: Triple-interlocked-nanotwinned bulk magnesium alloys with exceptional strength and ageing resistance
Source: Nat Commun. 2026 May 22;17:4775. doi: 10.1038/s41467-026-73640-w (PMC13219722; doi:10.1038/s41467-026-73640-w)
Supplement: Supplementary file 1 — Supplementary Information [file 41467_2026_73640_MOESM1_ESM.pdf]

Supplementary information

Triple-interlocked-nanotwinned bulk magnesium alloys with exceptional strength and ageing resistance

Qiuming Peng[1, *, †], Lutong Zhou[1, †], Jinming Wang[1, †], Ke Tong[1], Wentao Hu[1], Lin Wang[1], Yong Sun[1], Yipeng Gao[2], Anmin Nie[1], Biaobiao Yang[3, 4], Wei Cai[5], Guodong Zou[1, *], Tianlin Huang[5, *], Wanquan Zhu[5], Yongjun Tian[1, *]

[1]State Key Laboratory of Metastable Materials Science and Technology, Yanshan University, Qinhuangdao, 066004, P.R.China

[2]Key Laboratory of Automobile Materials of Ministry of Education & School of Materials Science and Engineering, Nanling Campus, Jilin University, Changchun 130025, P.R.China

[3]IMDEA Materials Institute, C/Eric Kandel 2, Getafe, 28906 Madrid, Spain

[4]Department of Materials Science, Polytechnic University of Madrid, Universidad Politécnica de Madrid, E.T.S. de Ingenieros de Caminos, 28040 Madrid, Spain

[5]International Joint Laboratory for Light Alloys, College of Materials Science and Engineering, Chongqing University, Chongqing 400044, P.R.China

[†]Authors with equal contributions to this work

[*]Corresponding authors: Qiuming Peng, pengqiuming@ysu.edu.cn;

Guodong Zou, zouguodong@ysu.edu.cn;

Tianlin Huang, huangtl@cqu.edu.cn;

Yongjun Tian, fhcl@ysu.edu.cn

The PDF file includes: Supplementary Figures. 1 to 27

Supplementary Notes 1 to 7

Supplementary Tables 1 to 6

Supplementary References

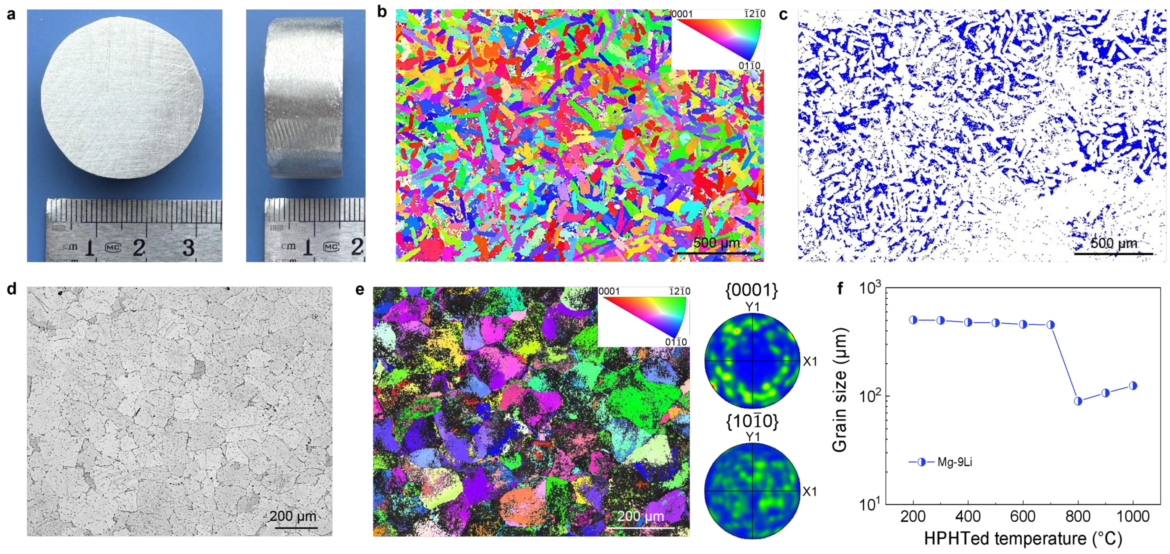

Supplementary Figure 1 | Microstructure of Mg-9Li samples. a, Surface morphology and dimension of HPHT samples. The dimeter and height are 30 mm and 15 mm, respectively. **b-c**, IPF image and the distribution of β-Li phase (blue) of the as-cast sample. The indexing rate is 90.47%. **d-e**, Optical structure, IPF image, and pole figures of the HPHT-800 sample. The indexing rate is 60.35%. The pole figures indicate that the HPHT-800 sample have no obvious texture. **f**, Grain size variation of the different HPHT Mg-9Li samples. Source data for **(f)** are provided as a Source Data file.

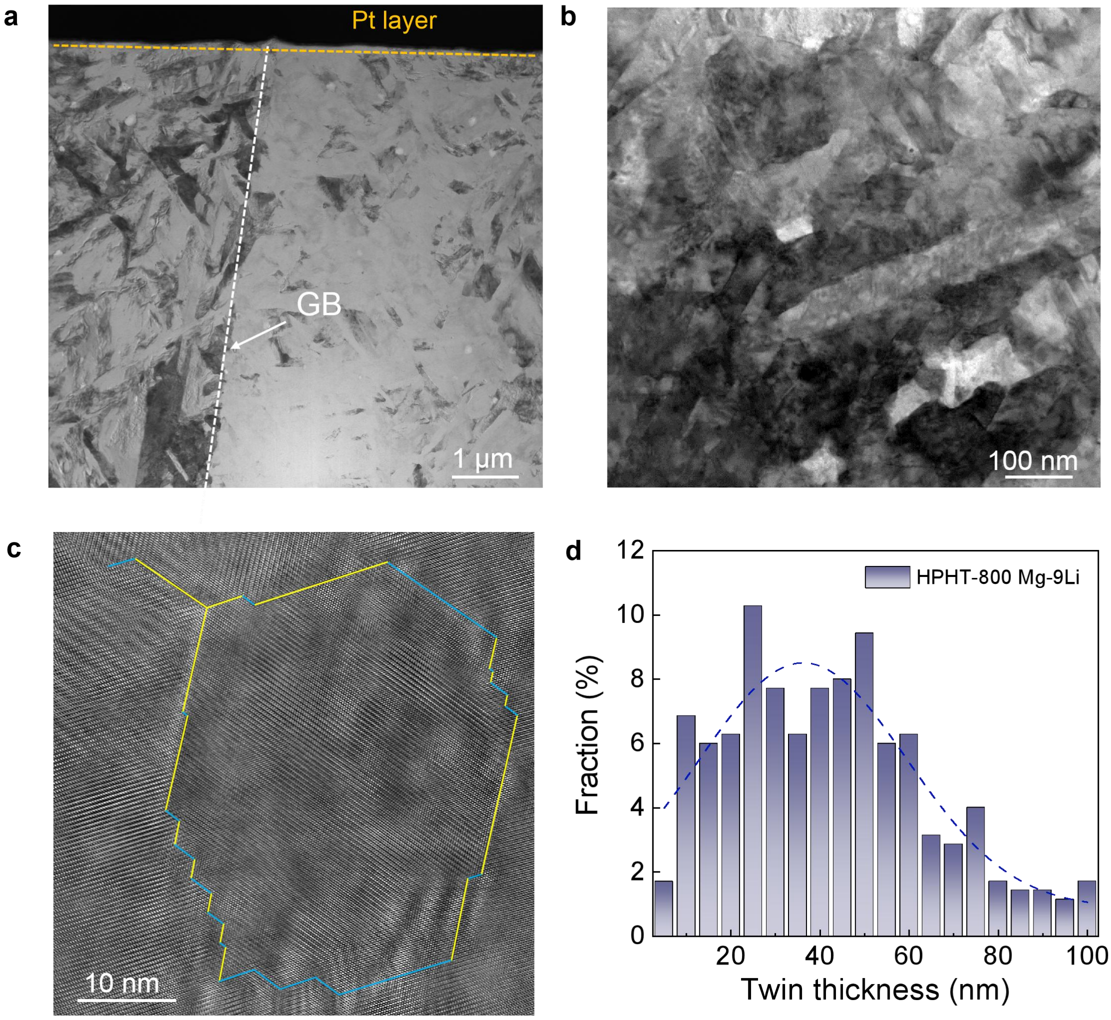

Supplementary Figure 2 | Interface characteristics. a, Low-magnification BF-TEM image of HPHT-800 Mg-9Li alloy. **b**, High-magnification BF-TEM image for twin-thickness investigation. **c**, HRTEM image of typical nanosized interface. **d**, Statistic distribution of twin thickness based on 100 random values. The average twin thickness is approximately 47±10 nm. Source data for **(d)** are provided as a Source Data file.

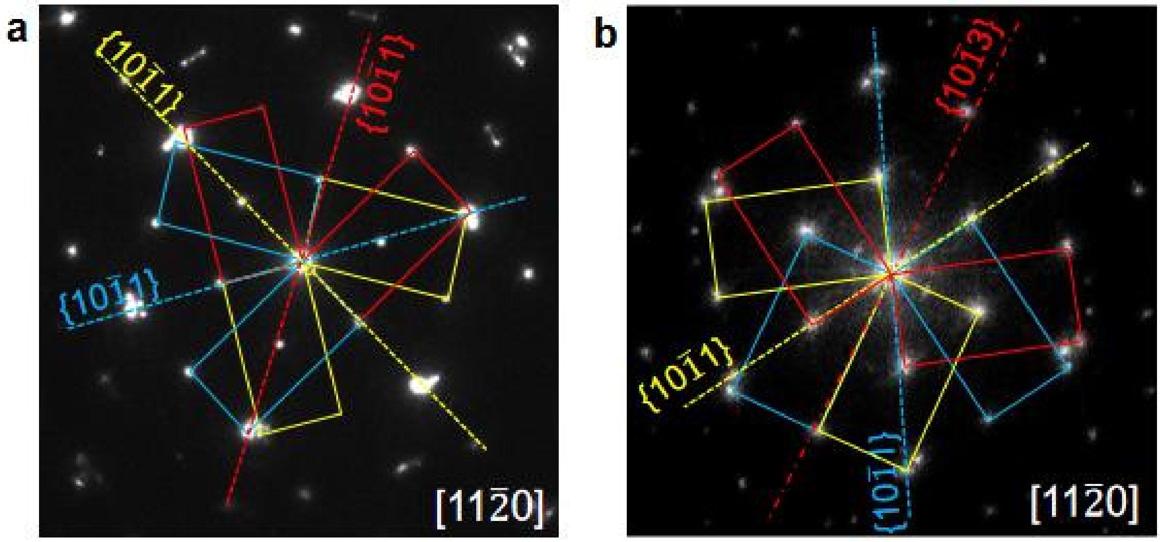

Supplementary Figure 3 | SAED patterns. The SAED patterns of i-TIT (**a**) and a-TIT (**b**) structures viewed along [11$\bar{2}$0], respectively.

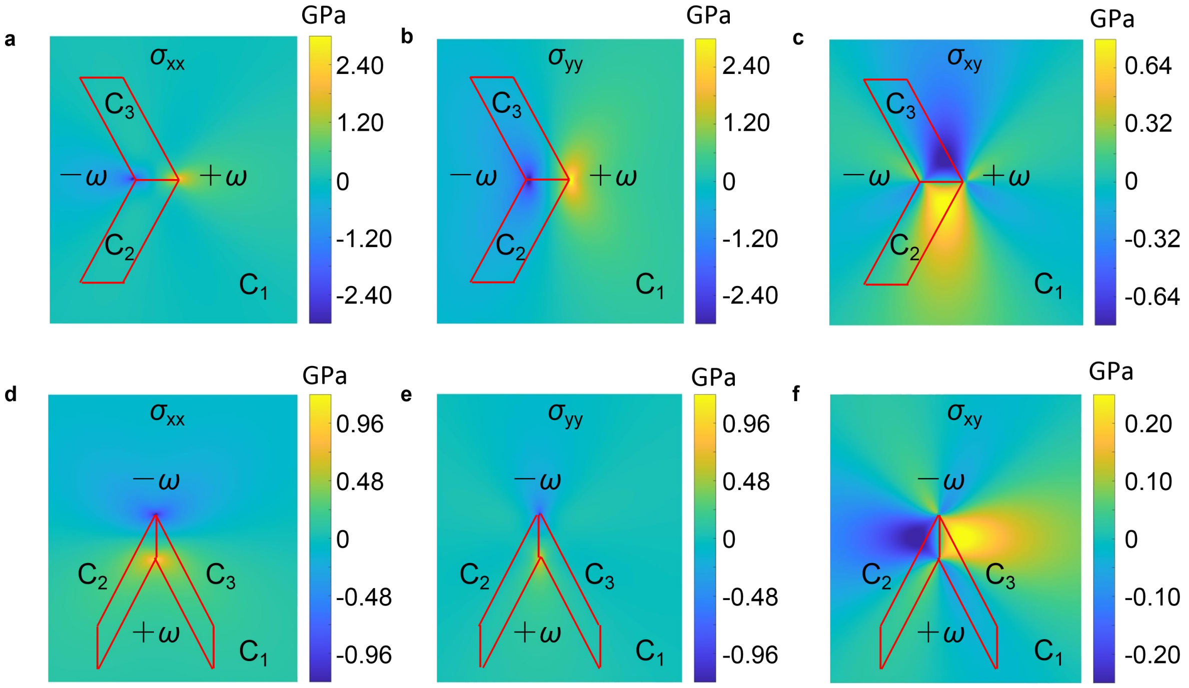

Supplementary Figure 4 | Structural stress analysis. **a-c**, Stress fields of the intersections among C_2 and C_3 twins in i-TIT structure. **d-f**, Stress fields of the intersections among C_2 and C_3 twins in a-TIT structure. The stress components include σ_{xx}, σ_{yy} and σ_{xy}.

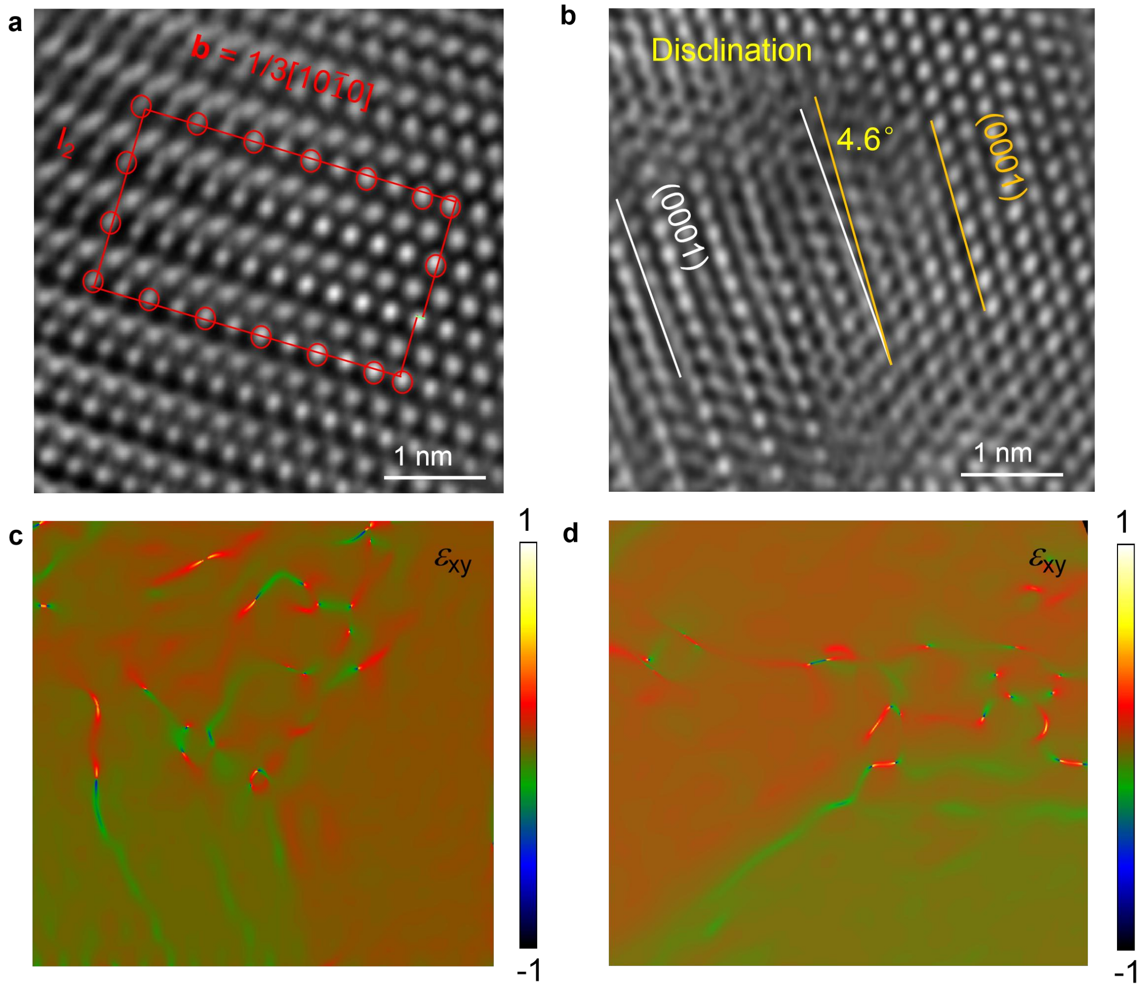

Supplementary Figure 5 | Interface characteristics. **a**, HRTEM image of basal intrinsic I_2-type stacking faults around {10 $\bar{1}$ 1} TBs viewed along [11 $\bar{2}$ 0]. Burgers vectors **b** of Shockley partial dislocation forming I_2-type SFs determined by the Burgers circuits drawn in (a). **b**, HRTEM image of a disclination with an angle of 4.6° near the TIT structure. **c** and **d**, GPA maps (in-plane rigid body rotation, ε_{xy}) correspond to the HRTEM images in Fig. 2b and Fig. 2c, respectively.

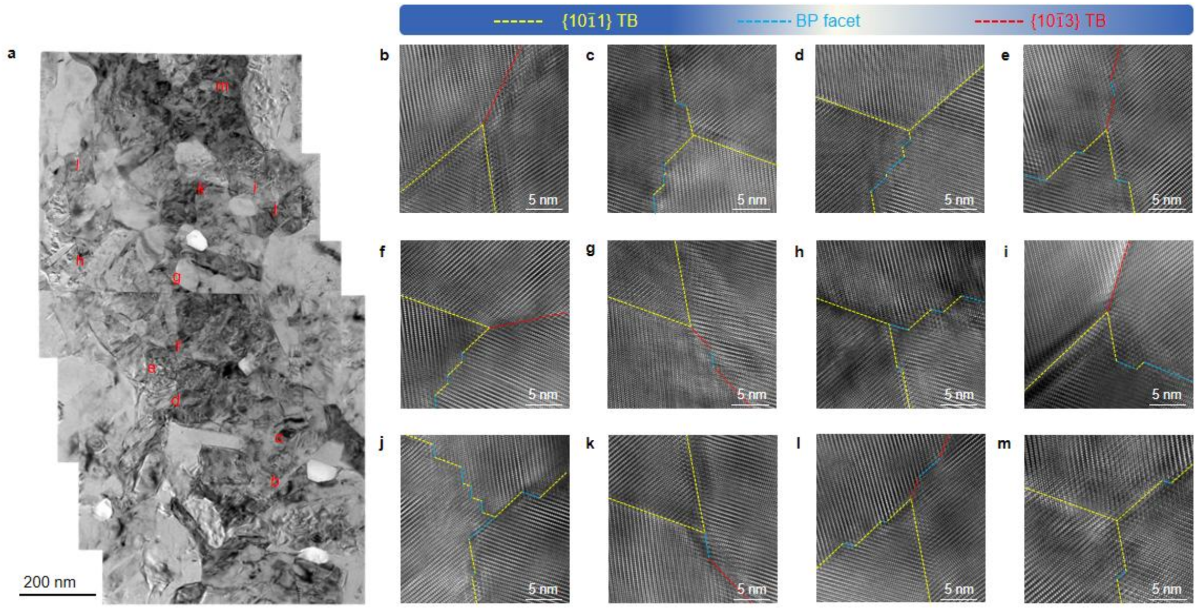

Supplementary Figure 6 | Homogenous distribution characteristics of TITs. a, BF-TEM image of TIT structures in an HPHT-800 Mg-9Li alloy viewed along [11$\bar{2}$0]. **b-m**, HRTEM images taken from the randomly selected fields of view within the red square regions in a. The viewing direction is parallel to $[11\bar{2}0]_{\alpha}$. The yellow dashed lines, red dashed lines, and blue dashed lines represent {10$\bar{1}$1} TBs, {10$\bar{1}$3} TBs, and BP facets, respectively.

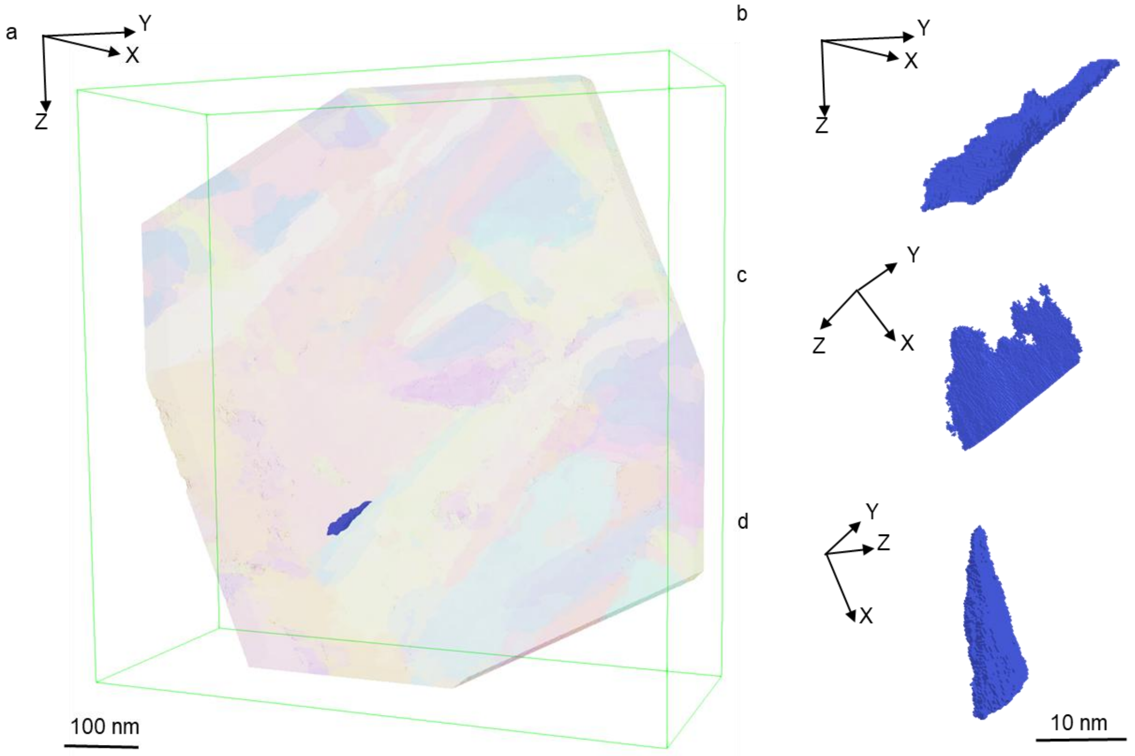

Supplementary Figure 7 | Spatial characteristics of TBs. a, A nanotwin lamella with an average thickness of ~13 nm embedded within the TIT structure. To highlight the nanotwin, the surrounding grains, coloured according to the IPF along Z direction, are rendered semi-transparent. **b-d**, 3D morphology of the nanotwin revealed by the reconstructed volume, shown from different viewing perspectives.

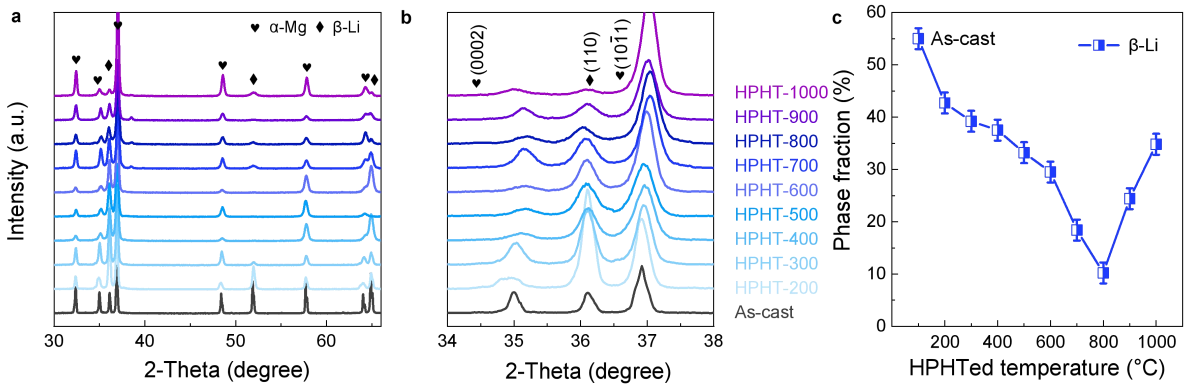

Supplementary Figure 8 | Phase composition. a, XRD patterns of HPHT Mg-9Li alloys under 6 GPa at different temperatures. **b**, Local high-magnification XRD patterns in the range of 34-38°. **c**, The phase fraction of the BCC β-Li phases under different HPHT conditions in terms of Rietveld refinement. Error bars represent the standard deviation derived from three independent experiments. Source data are provided as a Source Data file.

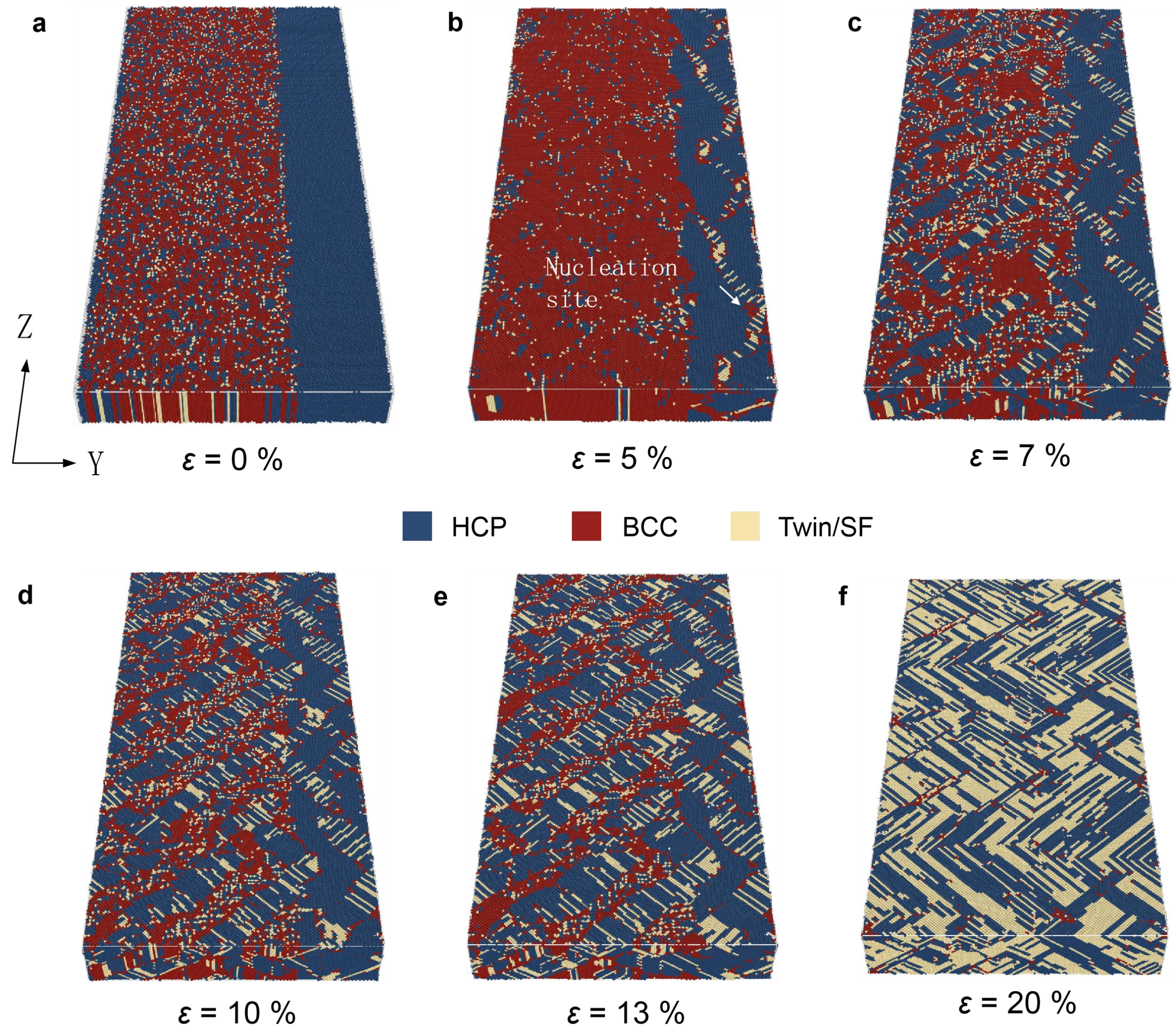

Supplementary Figure 9 | Microtructural evolution during formation process. a, Original Mg-9Li supercell. The blue region represents α-Mg with a HCP structure, the claret region represents β-Li with a BCC structure, and the yellow region represents α-Mg containing TB/SF structure. **b**, Two $\{10\bar{1}1\}$ twin variants are observed to nucleate from BCC nucleation sites in the HCP region when three-dimensional deformation is applied at 0 K. **c-f**, Twin variants gradually nucleate and grow as the strain increases. The MD data are provided at https://doi.org/10.24435/materialscloud:rf-56.

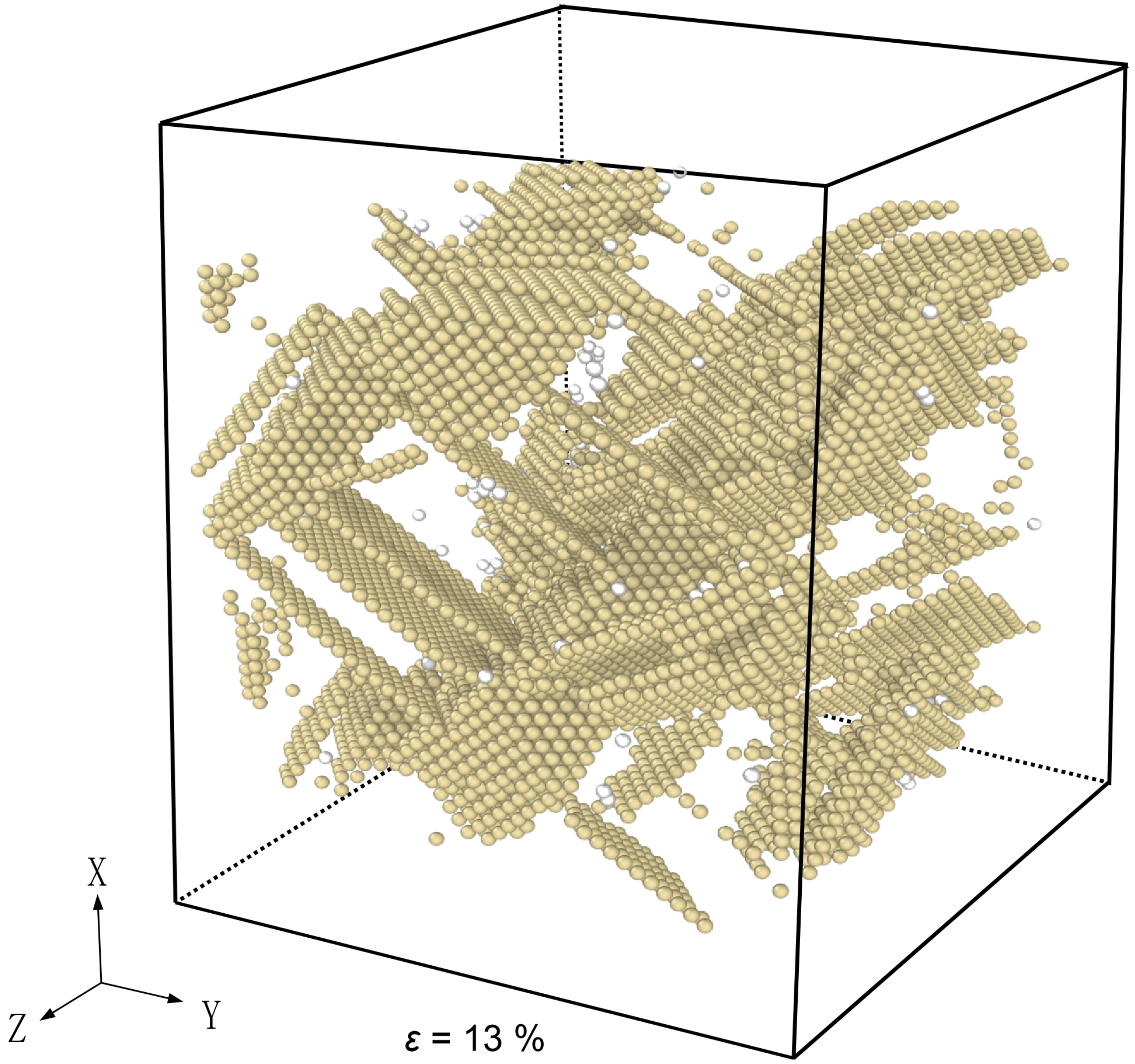

Supplementary Figure 10 | 3D structure of TIT. The typical net-shaped structure of TIT interface after removing the HCP/BCC matrix in deformed Mg-9Li alloy. The MD data are provided at https://doi.org/10.24435/materialscloud:rf-56.

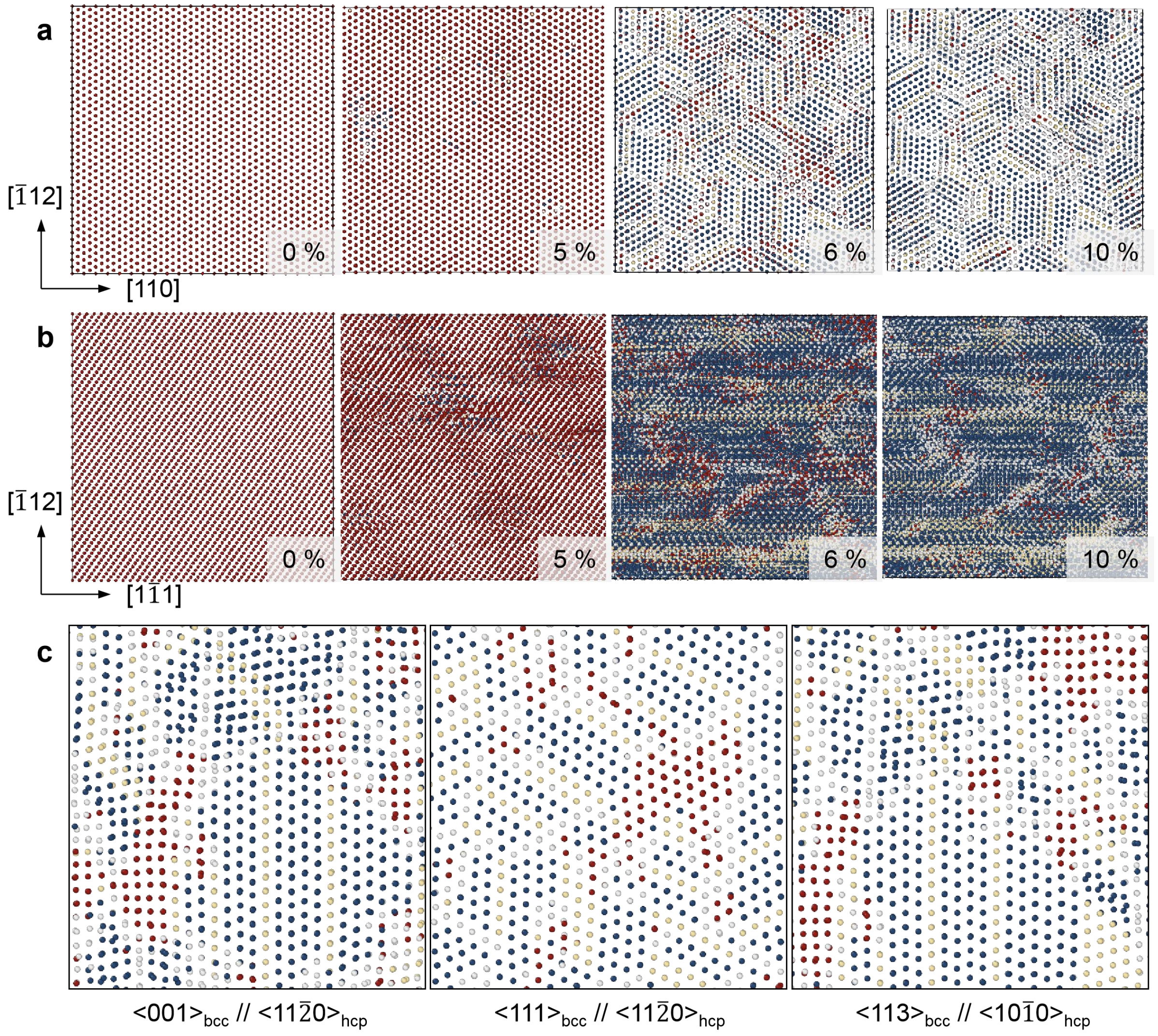

Supplementary Figure 11 | Crystallographic relationship of Martensitic transformation. Tensile deformation was performed along the $[1\,\bar{1}\,1]_{BCC}$ direction at 0 K. The blue region represents α-Mg with an HCP structure, the red region represents β-Li with a BCC structure, and the yellow region represents α-Mg that contains SF structure. **a**, BCC structure in Mg-9Li supercell viewed along $[1\bar{1}1]_{BCC}$. HCP phase begins to nucleate when the strain reaches 5 %. Subsequently, HCP variants with different orientations gradually interact and form twin structures as the strain increased to 6%. Finally, complete TIT structure has been formed as the strain increased to 10%. **b**, The same results also have been viewed along $[113]_{BCC}$ direction. **c**, The crystallographic relationship of martensitic transformation viewed along different directions. The MD data are provided at https://doi.org/10.24435/materialscloud:rf-56.

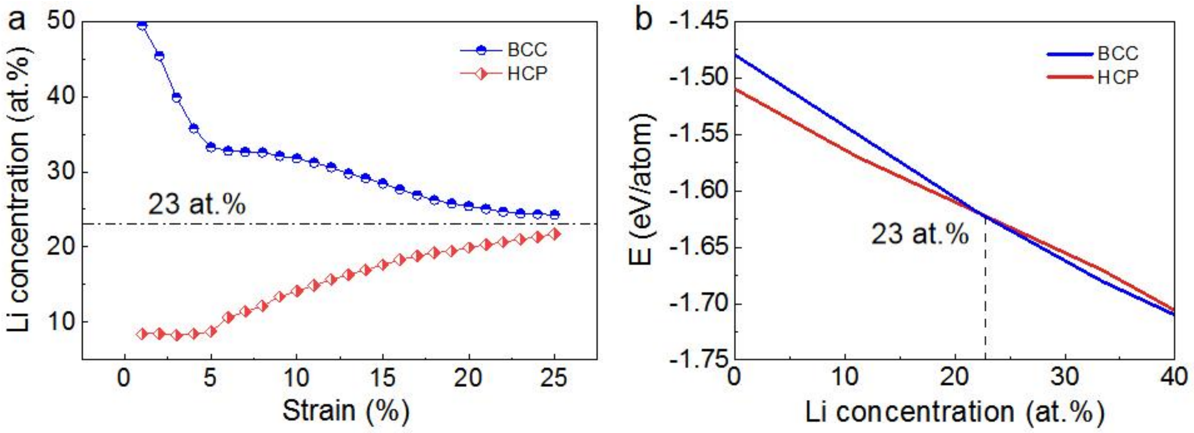

Supplementary Figure 12 | Martensitic transformation conditions. a, Li concentration variation in α-Mg and β-Li phases during MD simulation process. **b**, The variation curve between the energies of HCP and BCC structures dependent on the concentration of Li. The critical Li concentration is 23 at.%. The DFT data for (**b**) are provided at https://doi.org/10.24435/materialscloud:rf-56. Source data are provided as a Source Data file.

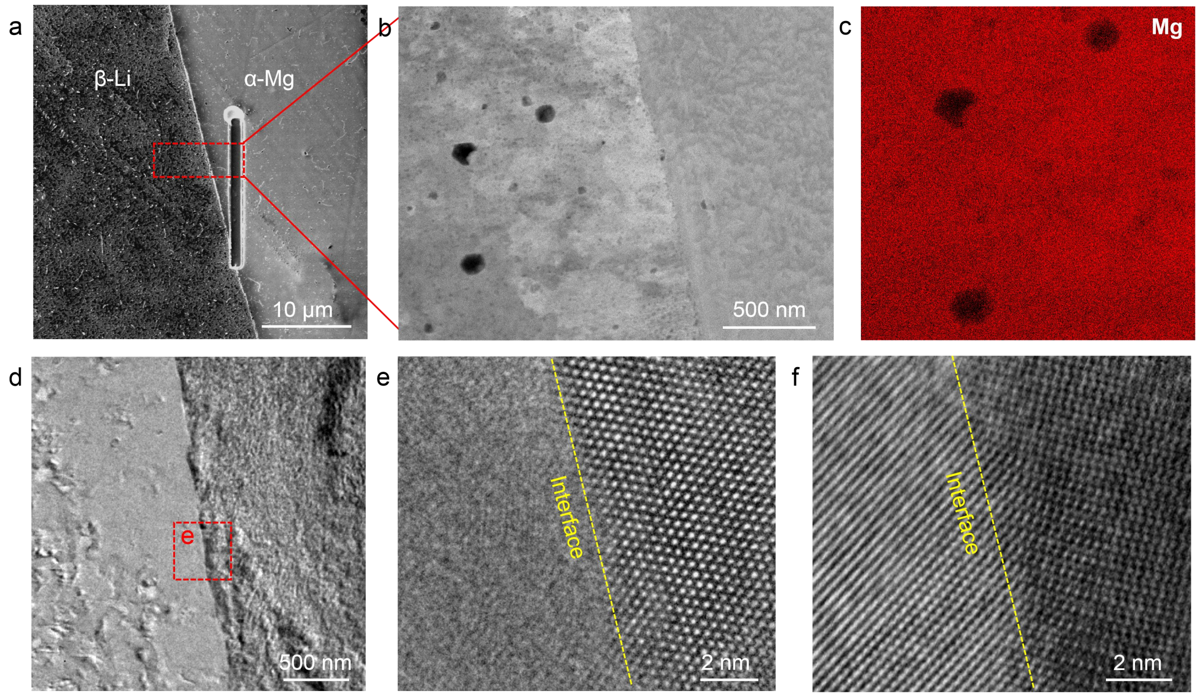

Supplementary Figure 13 | Microstructure of as-cast Mg-9Li alloy. a, SEM image of the interface of primary α-Mg and β-Li in as-cast Mg-9Li alloy. **b**, HAADF-STEM image of the interface structure. The left side is the β-Li region, and the right side is the α-Mg region. **c**, EDS mapping of the β-Li region. The results indicate that the black particles in (b) are Li-rich particles. **d**, Low magnification BF-TEM image of the interface structure. **e-f**, HRTEM images of the interface structure viewed along $[0001]_{\alpha}$ and $[11\bar{2}0]_{\alpha}$ direction, respectively. The results indicate that no crystallographic orientation relationship exists between α-Mg and β-Li phase in as-cast Mg-9Li sample.

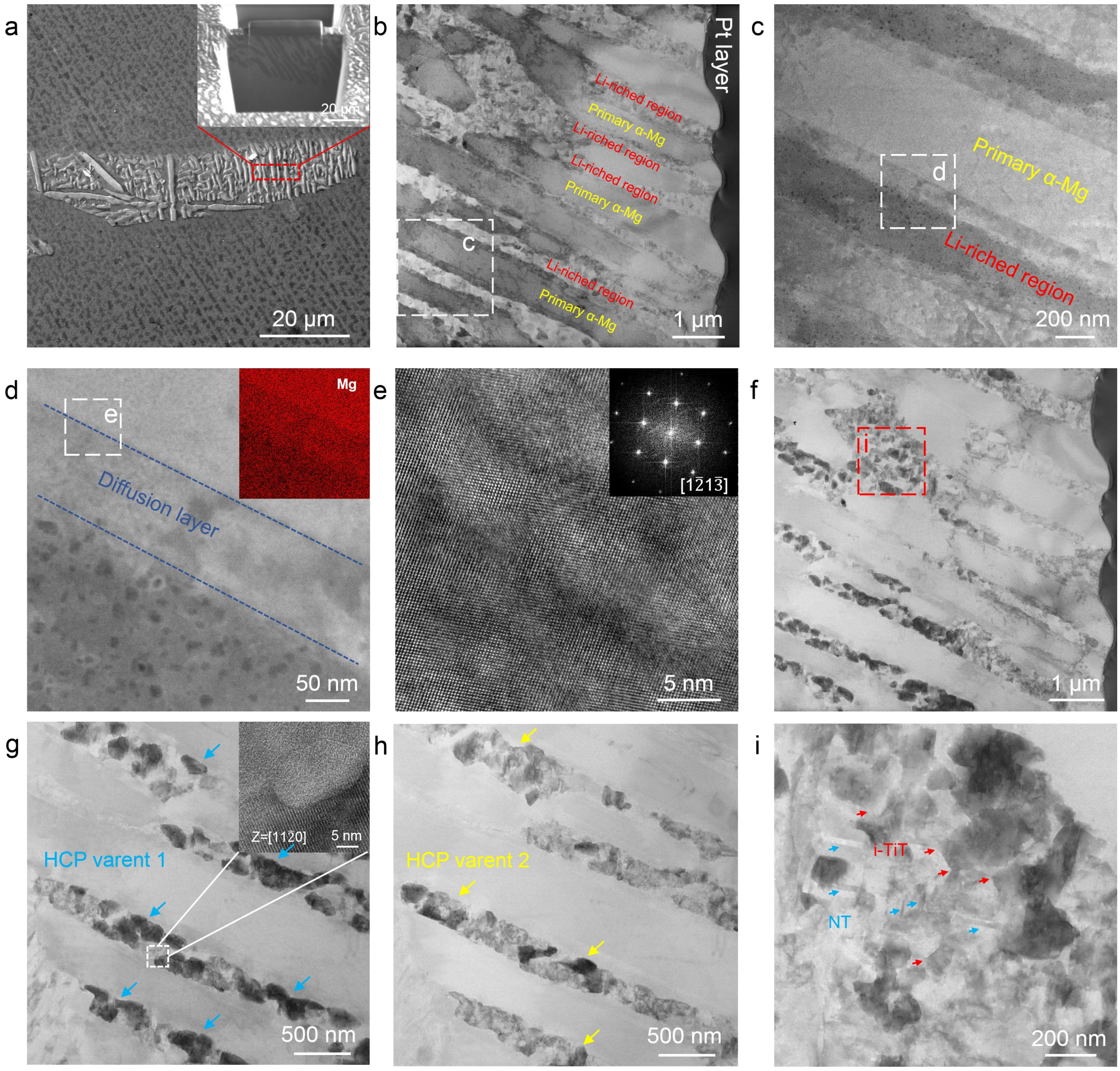

Supplementary Figure 14 | Microstructure of HPHT-500 Mg-9Li alloy. a, SEM image of the HPHT-500 Mg-9Li alloy. **b**, BF-TEM image of the primary α-Mg region viewed along $[1\bar{2}1\bar{3}]$ axis. **c**, HAADF-STEM image corresponding to the dashed box in b. **d**, High-magnification HAADF-STEM image of the diffusion layer. The inset shows the corresponding EDS mapping. **e**, HRTEM image of the diffusion layer viewed along $[1\bar{2}1\bar{3}]$. **f**, BF-TEM image of the Li-riched layer viewed along $[11\bar{2}0]$ axis. **g**, Local magnification image of f. **h**, BF-TEM image of the Li-riched layer viewed along $[11\bar{2}0]$ axis of the other HCP variant. **i**, High-magnification BF-TEM image of the interaction region between two Li-rich layers.

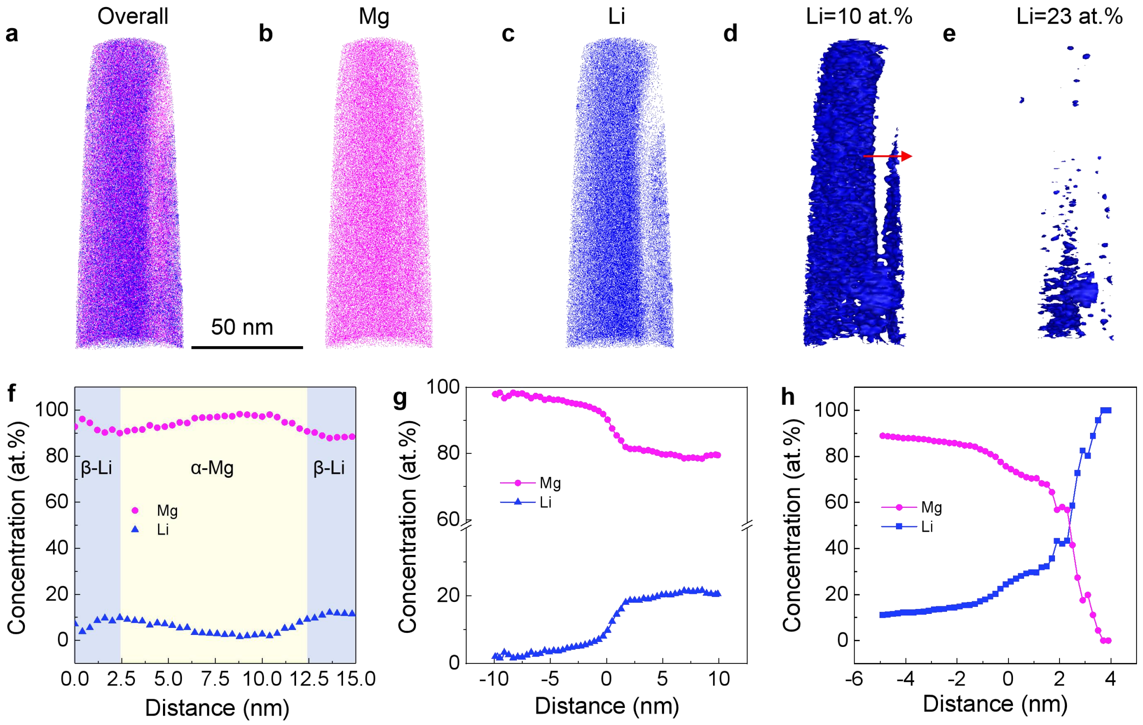

Supplementary Figure 15 | APT anaylsis of as-cast sample. a-c, Reconstructed APT volume showing the distribution of Mg and Li atoms. **d-e**, APT results showing the distribution of Li atoms defined by 10 at.% and 23 at.% Li iso-surfaces, respectively. **f**, Concentration profile along the red arrow in **d**. **g-h**, Concentration profiles corresponding to 10 at.% and 23 at.% Li iso-surface. Source data for **(f-h)** are provided as a Source Data file.

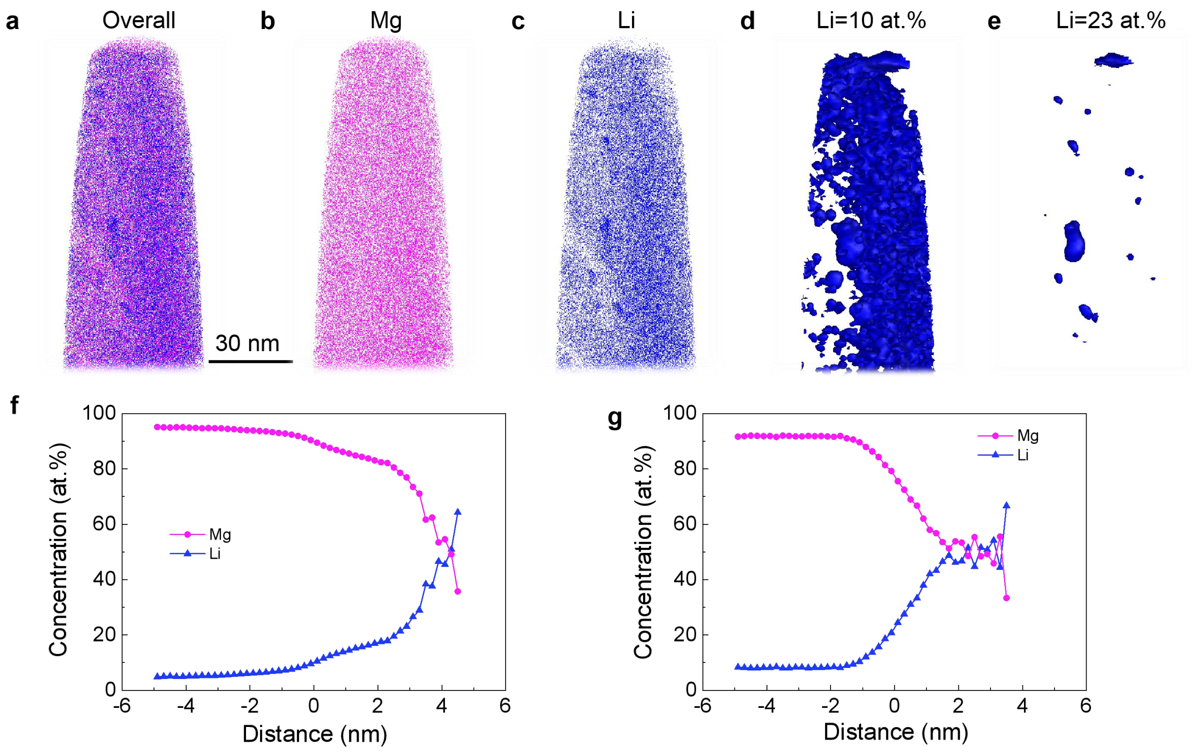

Supplementary Fig. 16 | APT anaylsis of HPHT-800 sample. a-c, Reconstructed APT volume showing the distribution of Mg and Li atoms. **d-e**, APT results showing the distribution of Li atoms defined by 10 at.% and 23 at.% Li iso-surfaces, respectively. **f-g**, Concentration profiles corresponding to 10 at.% and 23 at.% Li iso-surfaces, respectively. Source data for **(f-g)** are provided as a Source Data file.

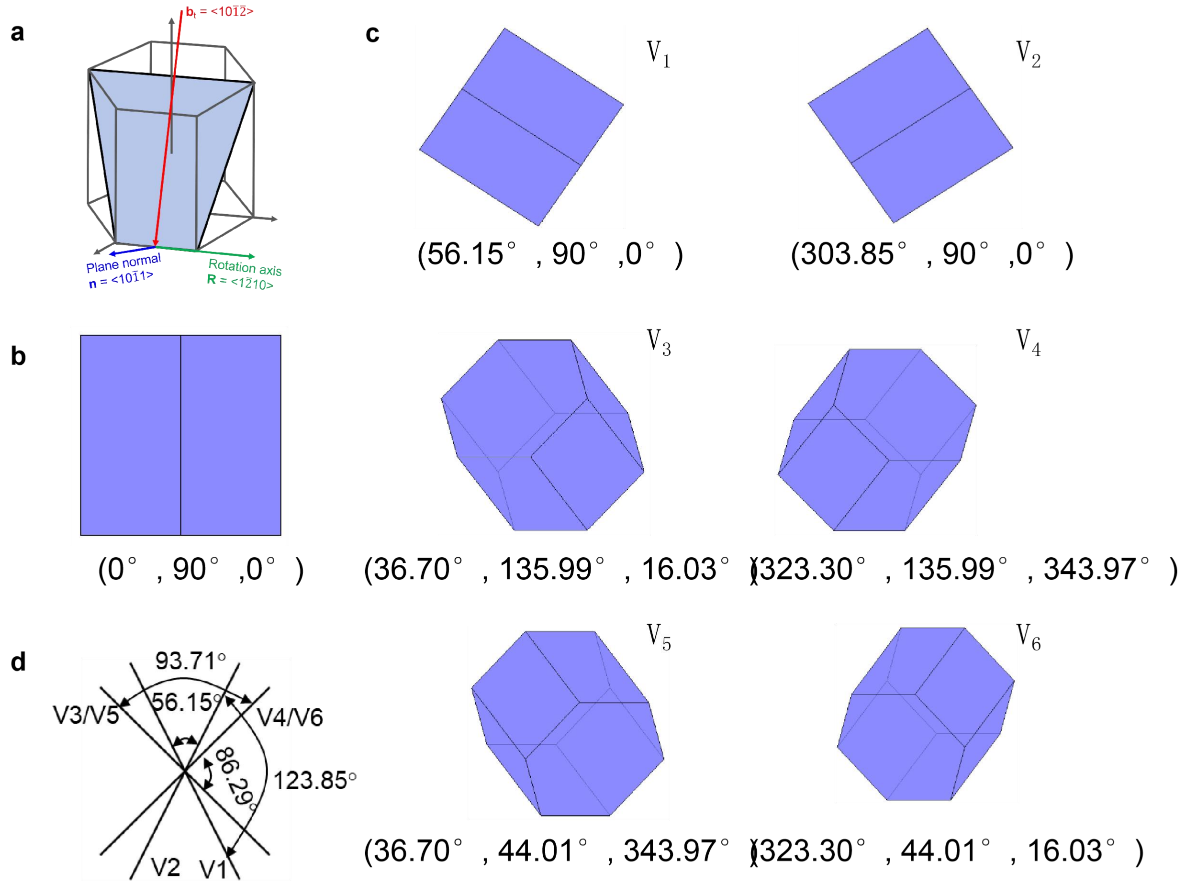

Supplementary Figure 17 | Spatial analysis of twin planes. a, The plane and orientaion of $\{10\bar{1}1\}$ twin in pure Mg. **b**, Initial parent grain. **c**, Six possible contraction twins denominated as V_1 to V_6. **d**, Theoretical projections of contraction twins on the $(1\bar{2}10)_{Mg}$ plane of parent grains. The Bunge Euler angles of possible contraction twins are involved.

a

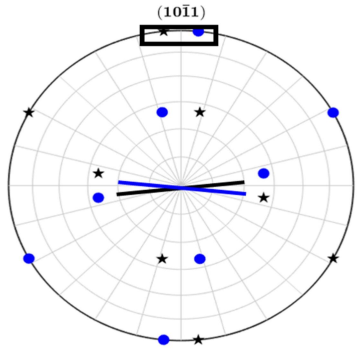

b

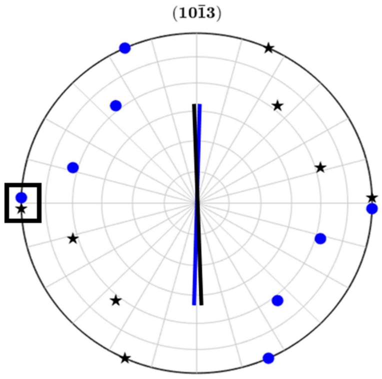

Supplementary Figure 18 | Pole figures of TIT structures. a, $\{10\bar{1}1\}$ pole figure of i-TIT structure. **b**, $\{10\bar{1}3\}$ pole figure of a-TIT structure. The stars and circles represent two different conjugated contraction twin variants, respectively. The bold lines stand for the nearly parallel low index lattice planes, corresponding to the projections outlined by the square of these two conjugated contraction twin variants.

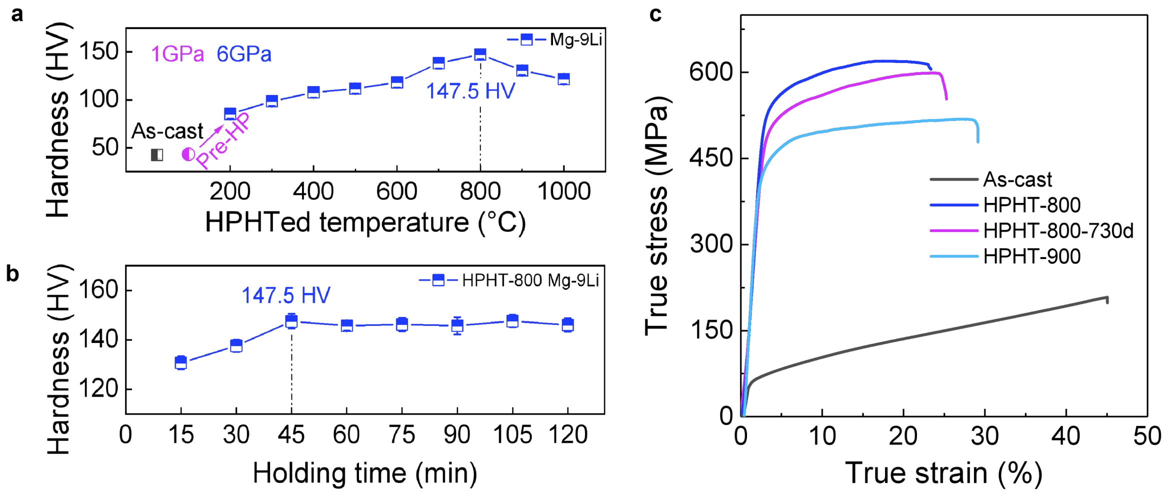

Supplementary Figure 19 | Vickers hardness and compression properties. **a**, Variations in Vickers hardness of Mg-9Li alloys as a function of high-pressure and high-temperature treatment. **b**, Variations in Vickers hardness of HPHT-800 Mg-9Li alloys as a function of holding time. **c**, Compressive true stress–strain curves of Mg-9Li alloys in various states at a nominal strain rate of 10^{-3} s^{-1}. Error bars in **a-b** represent the standard deviation of hardness derived from nine independent indentation points. Source data are provided as a Source Data file.

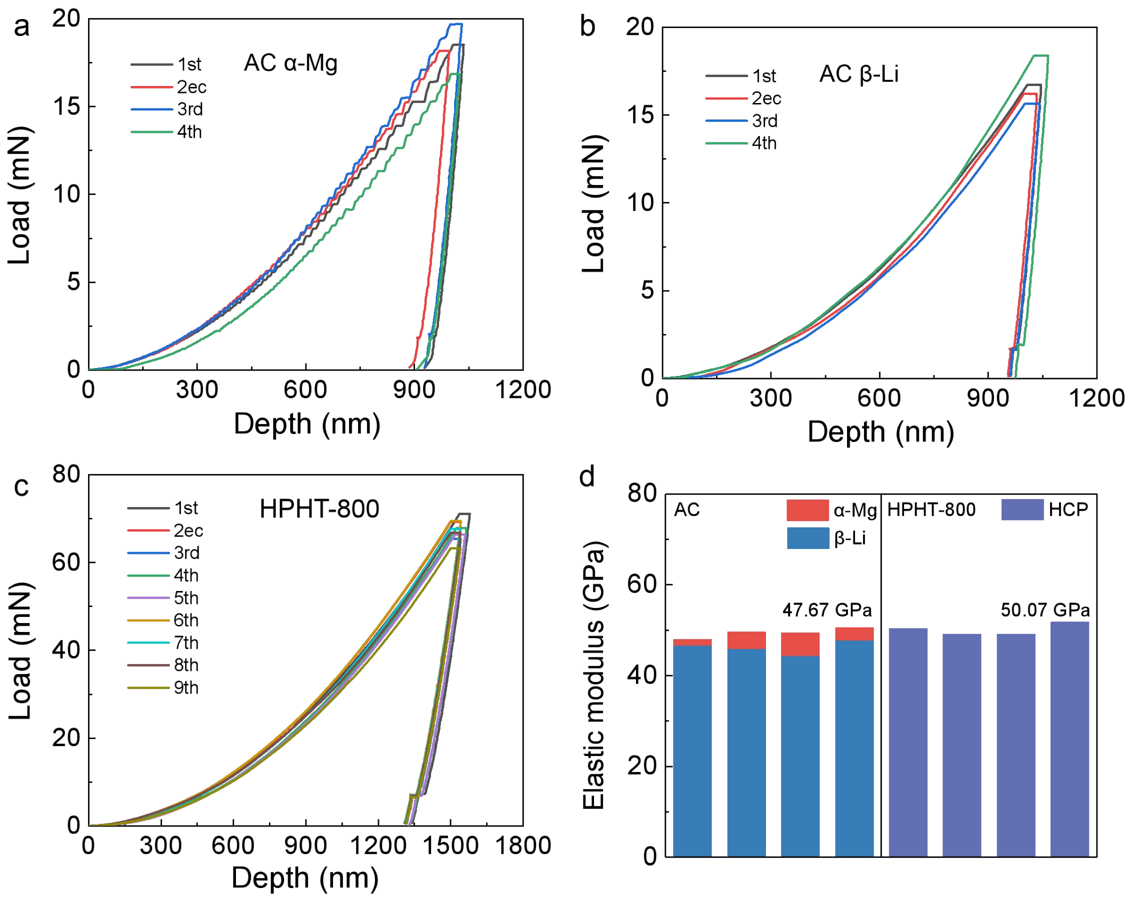

Supplementary Figure 20 | Elastic modulus of as-cast and HPHT-800 samples. a-b, The nanoindentation load-depth variation curves of the α-Mg and β-Li phases in the as-cast Mg-9Li sample respectively. **c,** The nanoindentation load-depth variation curves of the HPHT-800 Mg-9Li sample. **d**, The elastic modulus of as-cast and HPHT-800 samples obtained from the load-indentation depth curves. Source data are provided as a Source Data file.

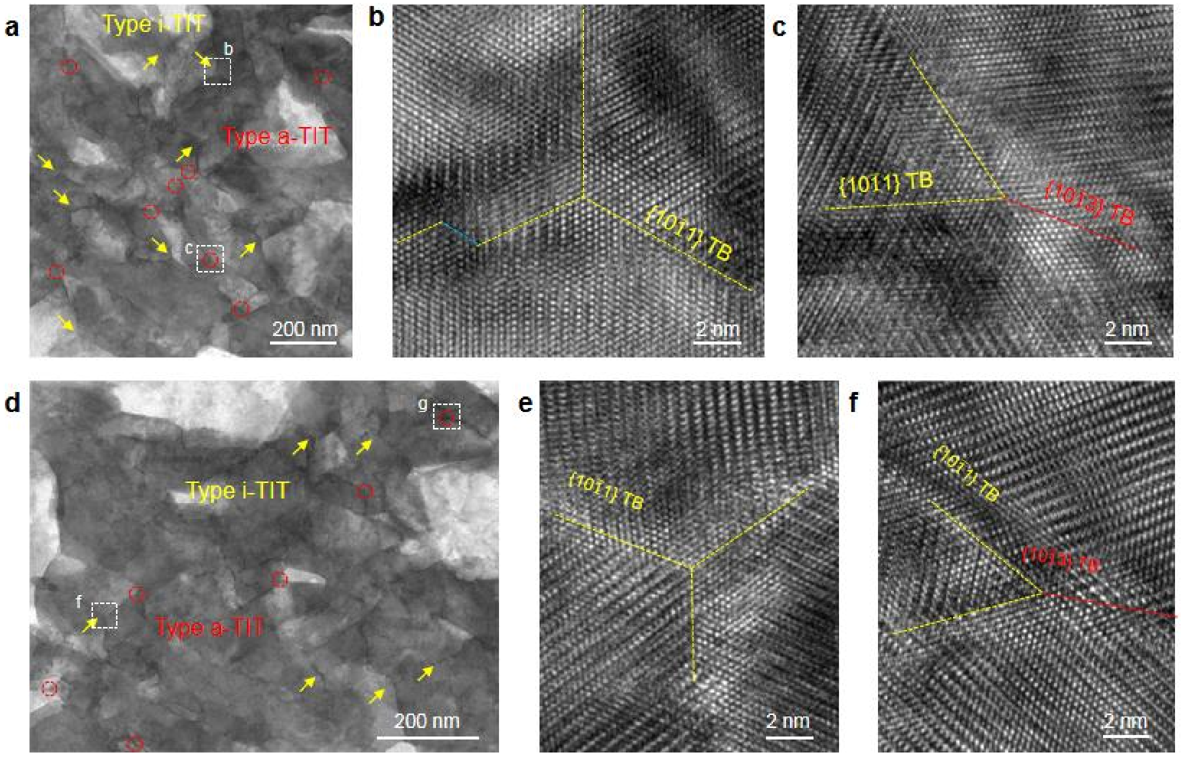

Supplementary Figure 21 | Long-term stability of TIT structures. a, BF-TEM image of the HPHT-800 Mg-9Li sample aged at ambient after two years. Based on statistical analysis, the average thickness of the nanotwin is 67 nm. **b-c**, HRTEM images of the i-TIT and the a-TIT structures in white dashed box indicated in **a**, respectively. **d**, BF-TEM image of the HPHT-800 Mg-9Li sample aged at 60 °C after 2000 h. Based on statistical analysis, the average thickness of the nanotwin is 78 nm. **e-f**, HRTEM images of the i-TIT and the a-TIT structures in white dashed box indicated in **d**, respectively.

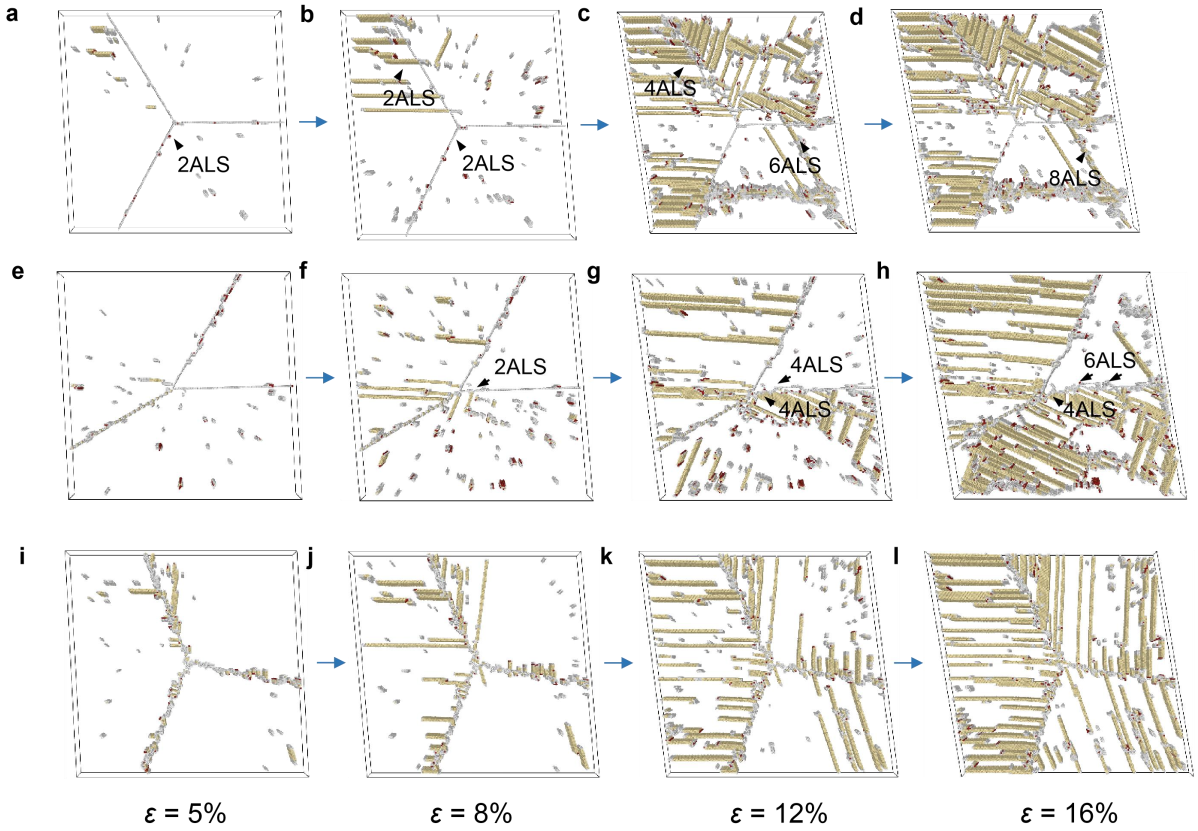

Supplementary Figure 22 | Deformation behaviour. a-l, MD simulations of the deformation processes of different structures. **a-d**, i-TIT supercell, **e-h**, a-TIT supercell and **i-l**, GB supercell, where each atomic layer is represented by ALS. Two-dimensional shear stress is applied in the horizontal direction at 0 K. The MD data are provided at https://doi.org/10.24435/materialscloud:rf-56.

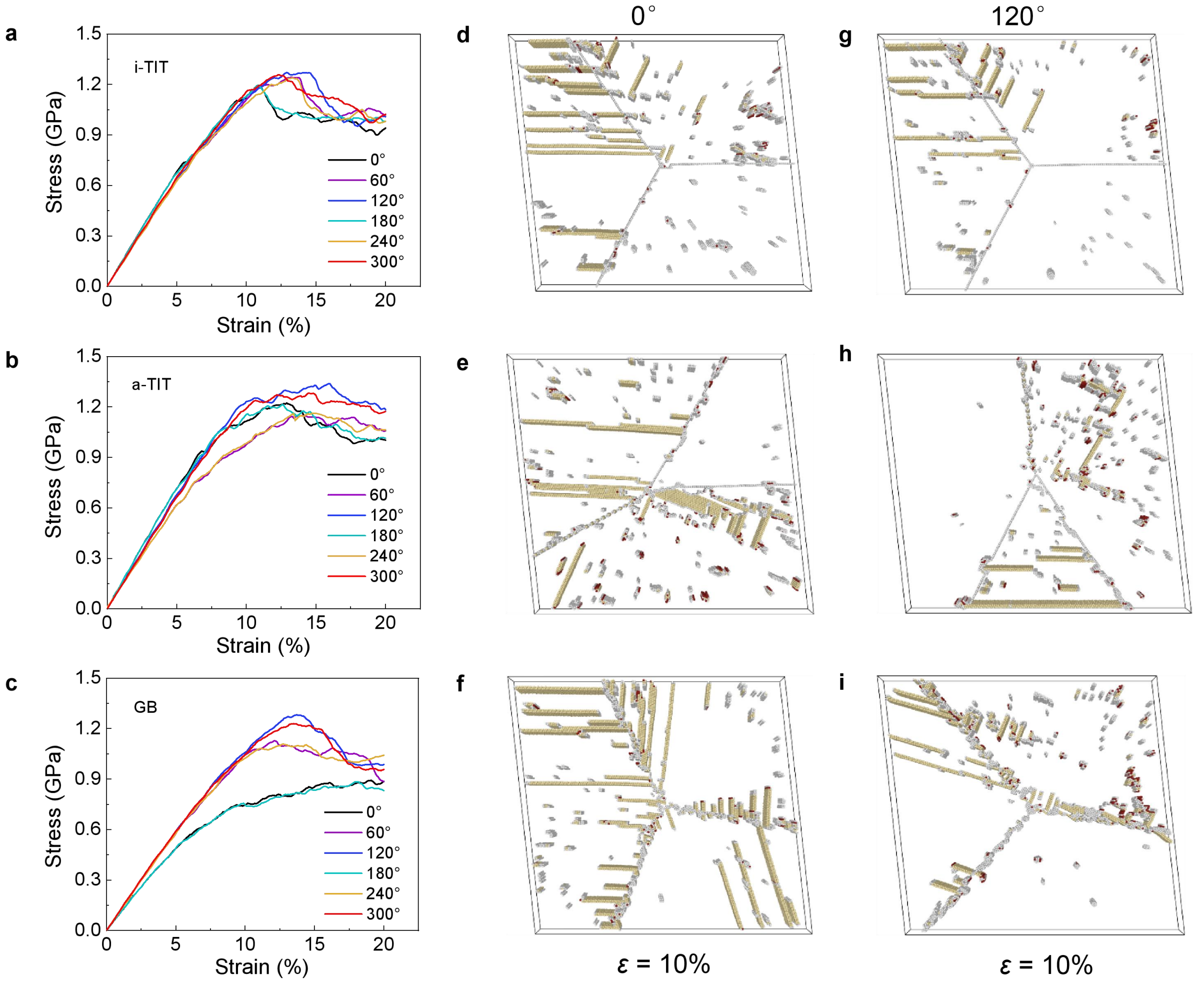

Supplementary Figure 23 | Strengthening anisotropy of different structures. a-c, Stress-strain curves of (a) i-TIT structure, (b) a-TIT structure and (c) GB structure simulated by MD, respectively. The relaxed structures were rotated by an increment of 60°, namely, 0°, 60°, 120°, 180°, 240°, and 300°. The shear stress is applied in the horizontal direction. Owing to the existance of large angle in incoherent GBs, it exhibits appearent mechanical anisotropy in contrast to other two TIT structures. **d-l**, The deformation morphology of i-TIT structure (d and g), a-TIT structure (e and h) and GB structure (f and i) rotate 0 and 120 degrees, respectively. The shear strain along the horizontal direction is 10%. The MD data are provided at https://doi.org/10.24435/materialscloud:rf-56. Source data for **(a-c)** are provided as a Source Data file.

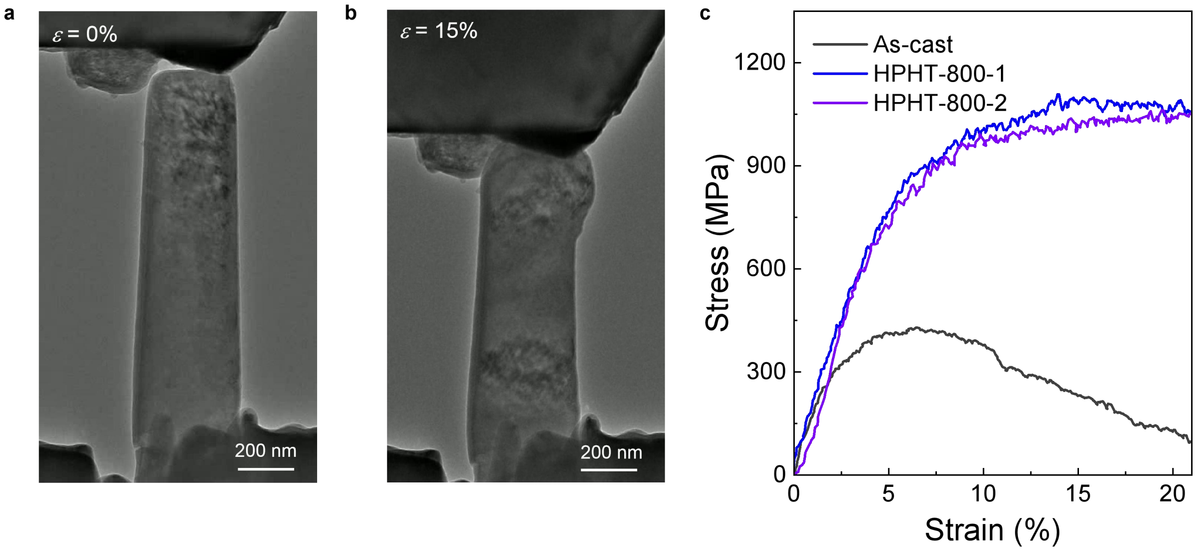

Supplementary Figure 24 | In situ deformation experiment of nanopillar. a and b, BF-TEM images of the HPHT-800 Mg-9Li nanopillar at strains of 0% and 15%, respectively. **c**, Compression test curves of as-cast and two HPHT-800 Mg-9Li nanopillars with different orientations. Source data for **(c)** are provided as a Source Data file.

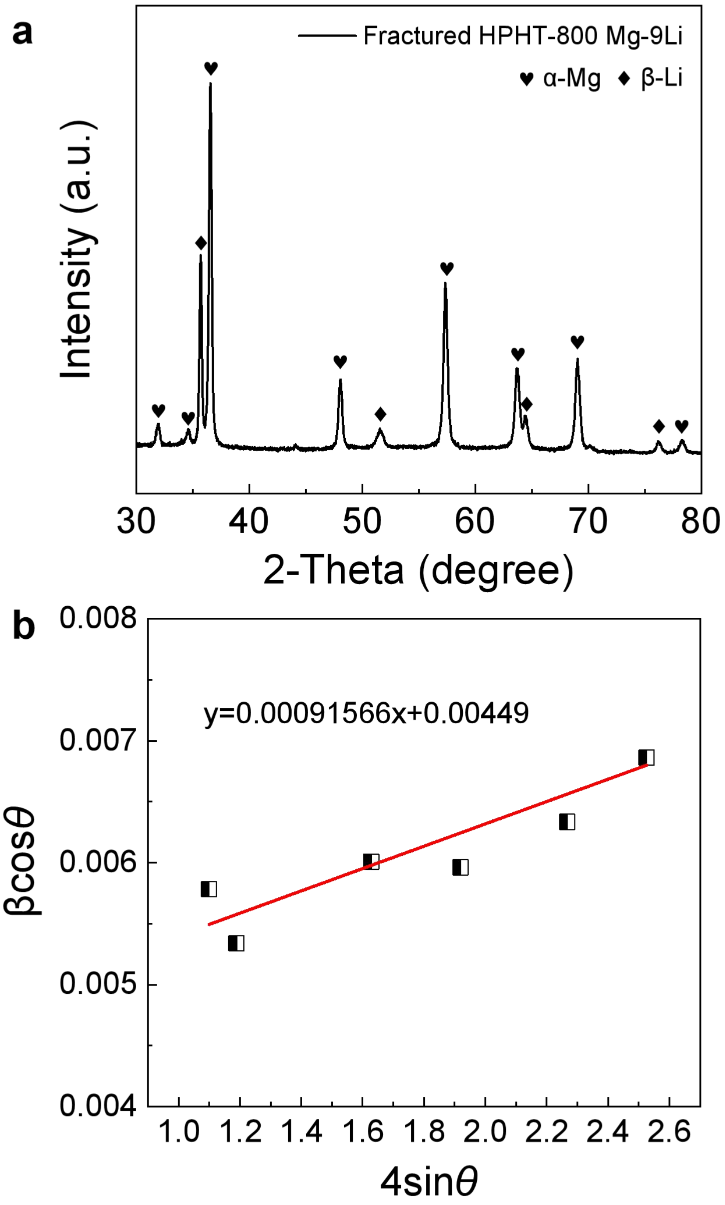

Supplementary Figure 25 | Dislocation density. a, XRD pattern of the HPHT-800 sample after tensile fracture. **b**, The fitted curve for the Classic Williamson-Hall (W-H) method. According to calculation, the dislocation density is approximately 1.171×10^{14} m^{-2}. Source data are provided as a Source Data file.

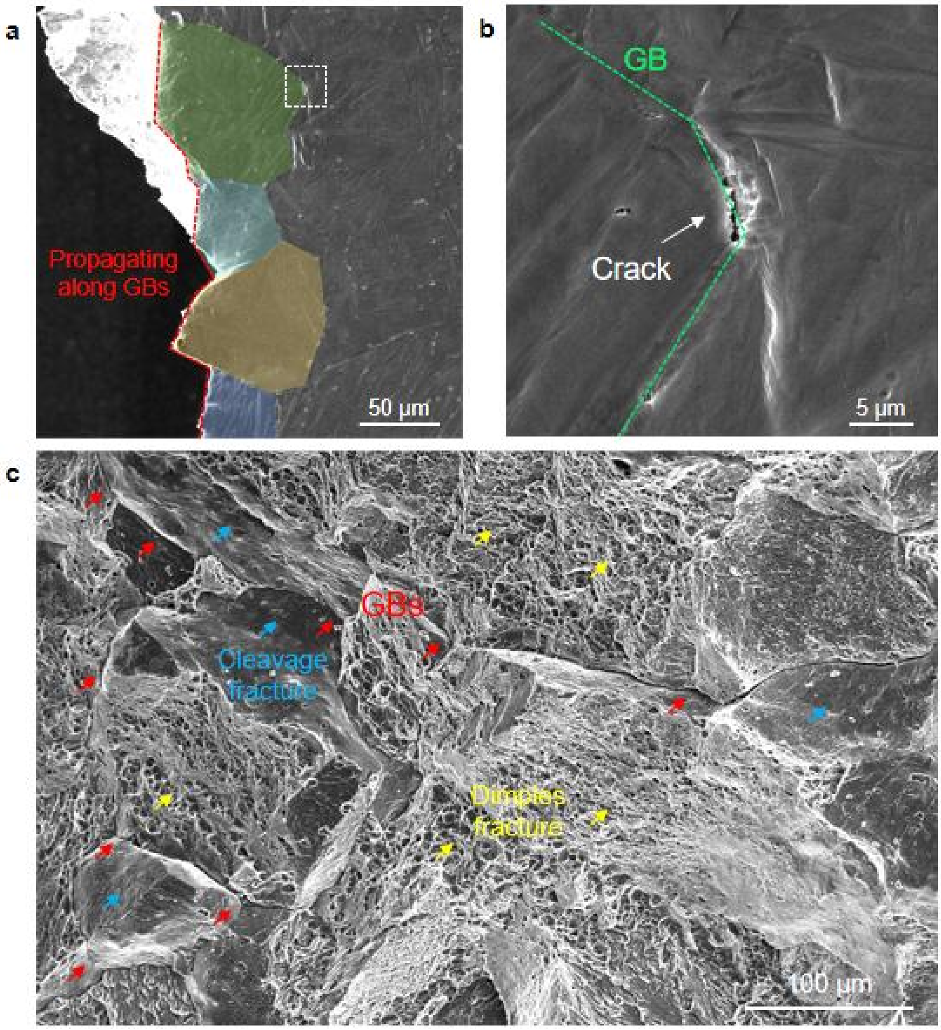

Supplementary Figure 26 | Fracture surface and crack propagation. a, SEM image of the fracture tip of HPHT-800 Mg-9Li alloy after tensile testing. **b**, High magnification SEM image of GB including crack. **c**, Fracture surface of HPHT-800 Mg-9Li alloys.

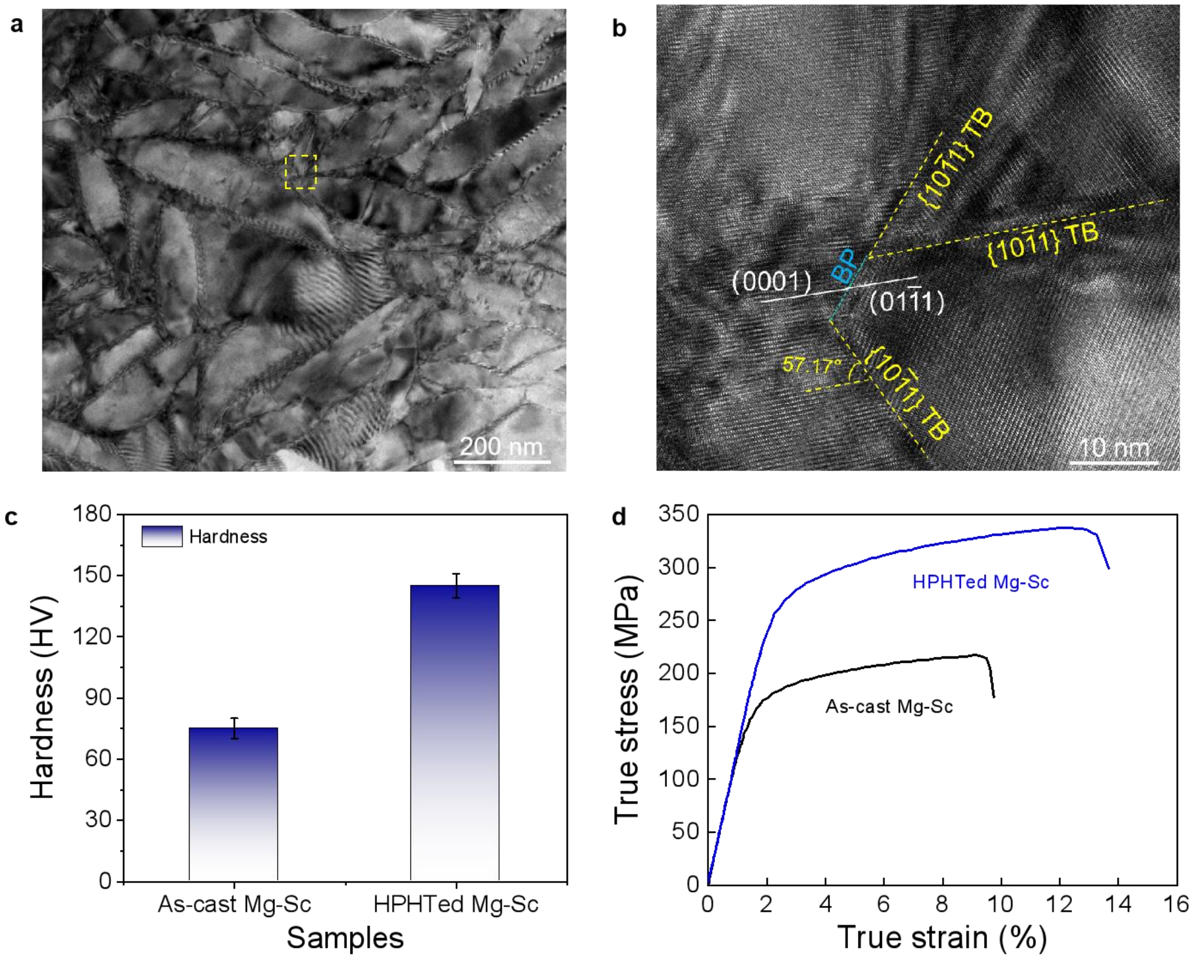

Supplementary Figure 27 | Microstructure and properties of Mg-Sc alloys. a, Low-magnification BF-TEM images of HPHTed Mg-Sc sample. **b**, HRTEM image of triple interlock interfaces, selected from dashed area in (a). **c**, Hardness values of the as-cast and HPHTed Mg-Sc samples, respectively. Error bars represent the standard deviation of hardness derived from nine independent indentation points. **d**, Comparison of tensile properties of the as-cast and HPHTed Mg-Sc samples. Source data for **(c-d)** are provided as a Source Data file.

Supplementary Note 1: Stress fields of the contraction twin–twin interaction

To determine the local stress field distribution surrounding a twin–twin intersection, analytical stress field expressions based on the defect configuration of the wedge disclination dipole can be employed as follows [1]:

$$\begin{cases} \sigma_{xx} = \dfrac{G\omega}{2\pi(1-\nu)} \cdot \left[\dfrac{1}{2}\ln\dfrac{x^2+(y+L)^2}{x^2+(y-L)^2} + \dfrac{x^2}{x^2+(y+L)^2} - \dfrac{x^2}{x^2+(y-L)^2}\right] \\ \sigma_{yy} = \dfrac{G\omega}{2\pi(1-\nu)} \cdot \left[\dfrac{1}{2}\ln\dfrac{x^2+(y+L)^2}{x^2+(y-L)^2} + \dfrac{(y+L)^2}{x^2+(y+L)^2} - \dfrac{(y-L)^2}{x^2+(y-L)^2}\right] \\ \tau_{xy} = \dfrac{G\omega}{2\pi(1-\nu)} \cdot \left[\dfrac{x(y+L)}{x^2+(y+L)^2} - \dfrac{x(y-L)}{x^2+(y-L)^2}\right] \\ \sigma_{zz} = \dfrac{G\omega\nu}{2\pi(1-\nu)}\ln\dfrac{x^2+(y+L)^2}{x^2+(y-L)^2} \end{cases} \quad (1)$$

where the variables x, y, and z correspond to the coordinates of the field in the Cartesian coordinate system O-xyz; ω is the magnitude of the Frank vector of the disclination; G is the shear modulus; and ν is Poisson's ratio. For the defect configuration in Fig. 2e and 2f , the disclination line was parallel to the z-axis, and the centre of the disclination dipole was located at y=0, with a positive disclination at $+\omega$ (0, -L) and a negative disclination at $-\omega$ (0, L). Here, for convenience, L was considered to be 1 μm. The local stress field distribution surrounded a twin–twin intersection of C_1-C_2-C_3 (Supplementary Fig. 4 and Supplementary Table 2), corresponding to the formation of an 11.6° wedge disclination dipole, with C2 and C3 corresponding to the $(1\bar{1}0\bar{1})_{C1}$ and $(1\bar{1}01)_{C1}$ twins, respectively. The calculated stress field of the C_1-C_2-C_3 twin reaction resulted in the formation of a 3.7° wedge disclination dipole (Supplementary Fig. 4 and Supplementary Table 2). Notably, there was a significant stress concentration surrounding the disclinations at the triple junction.

Supplementary Note 2: Conditions and process of martensitic-like transformation

The BCC→HCP microstructure evolution observed in this work is a martensitic-like transformation driven by compositional fluctuations. The specific conditions and mechanisms are as follows: Unlike conventional martensitic transformations driven directly by shear at low temperatures or under stress, this transformation is triggered by the diffusion and redistribution of Li atoms during the HPHT process. This argument is supported by XRD refinement results (Supplementary Fig. 8). If the transformation was only driven by shear, the BCC β-Li phase would be expected to transform into an HCP-structured Li phase. However, no corresponding diffraction signals for an HCP-Li phase were detected in XRD patterns. Furthermore, according to supplementary first-principles calculations (Supplementary Fig. 12), the free energies of the BCC and HCP phases intersect at a Li concentration of approximately 23 at.%. This implies that when the local Li concentration exceeds this critical value, the BCC phase is more stable. Conversely, when the concentration falls below this value, the system spontaneously transforms from BCC to HCP via a martensitic transformation. Meanwhile, we have quantified the Li concentration changes within two phases during the MD simulation process (Supplementary Fig. 12). Moreover, the reconstructed APT results indicated that the average Li concentrations are 3.3 at.% for the primary α-Mg phase and 20.2 at.% for the β-Li phase in the as-cast sample. In some local areas, the Li concentration is even over 50 at.% (Supplementary Fig. 15). In contrast, the analysis of distinct regions reveals an average Li concentration of 7.3 at.% for the Li-poor HCP variants and 14.5 at.% for Li-riched HCP variants in the HPHT-800 sample (Supplementary Fig. 16). Both experimental and simulation evidence further supports that the martensitic-like transformation is primarily driven by Li compositional fluctuations.

During the HPHT process, high temperature provides a significant driving force for Li diffusion, while high pressure likely further promotes Li redistribution by affecting the chemical potential gradient. This leads to compositional fluctuations within the initial β-Li phase, creating local Li-poor regions (< 23 at.%). Once this thermodynamic condition is met, these regions undergo a martensitic-like transformation, generating HCP. This process can be supported by the updated TEM data of the HPHT-500 sample (Supplementary Fig. 14). The

primary α-Mg regions and Li-rich regions are clearly distinguished by the HAADF-STEM image (the characteristic is consistent with the as-cast sample, Supplementary Fig. 13). A compositional diffusion layer exists between them, with no crystallographic difference observed (Supplementary Fig. 14b-e). More importantly, HCP variants with different orientations have begun to form within the Li-rich regions (Supplementary Fig. 14g-h). As the consumption of primary α-Mg regions, the HCP variants originating from different Li-rich regions interact with each other, ultimately forming the complex TIT network (Supplementary Fig. 14i). This direct observation provides key evidence for the continuous evolution process: "compositional fluctuation → local phase transformation → nucleation of multiple HCP variants → formation of the TIT network."

Moreover, the crystallographic orientation relationship of this martensitic transformation has been confirmed through updated MD simulations (Supplementary Fig. 11). We subjected the initial β-Li region in the Mg-9Li model to tensile deformation along different directions. The results show that at a strain of 6%, HCP variants begin to appear locally. When the strain reaches 10%, the initial β-Li phase is almost completely transformed into HCP variants of different orientations, which then assemble into a TIT structure. Analysis of the crystallographic orientation during the transformation reveals a $[1\ \bar{1}\ 1]_{BCC}$ // $[11\ \bar{2}\ 0]_{HCP}$ relationship, consistent with previously reported martensitic transformations in titanium. Therefore, this work reveals a "diffusion-assisted, compositional-fluctuation-induced martensitic-like transformation" mechanism. It differs from the traditional shear mechanism and highlights the prerequisite role of compositional fluctuations in initiating the phase transformation under high pressure.

Supplementary Note 3: Spatial configuration of the TIT structure

For the i-TIT, three TBs corresponding to the $\{10\bar{1}1\}$ lattice plane with intersection angles of approximately 124°, 118° and 118° were detected. For the a-TIT, two TBs corresponded to the $\{10\bar{1}1\}$ lattice plane with an intersection angle of 56°, and the other TB matched well with the $\{10\bar{1}3\}$ lattice plane, and the angle between this boundary and the other two boundaries was 152°. Note that the zone axis used to observe these microstructures via TEM was $[1\bar{2}10]_{Mg}$. Given that the sample was subjected to high pressure using a cubic press machine and that $\{10\bar{1}1\}$ boundaries were frequently observed in the postmortem microstructures, the $\{10\bar{1}1\}$ $\langle 10\bar{1}\bar{2}\rangle$ contraction twin (rotation axis: $\langle 1\bar{2}10\rangle$, rotation angle: 56.15°) should be relevant. Under the zone axis of $[1\bar{2}10]_{Mg}$, for a parent grain, e.g., with Bunge Euler angles of (0°, 90°, and 0°), all the possible contraction twins and the projections of their theoretical twinning planes on the $(1\bar{2}10)_{Mg}$ plane were calculated using MTEX (version 5.7.0), an open-source MATLAB toolbox [2], as shown in Supplementary Fig. 17. Note that the V1 and V2 contraction twin variants were conjugated, and their twinning planes were parallel to the paper normal. One interpretation of the i-TIT type was proposed here in which the two conjugated contraction twins impinge each other and constitute a unique microstructure together with residual parent grains (Fig. 4i). Note that their twinning shear directions were different, i.e., one belonging to $\langle 10\bar{1}\bar{2}\rangle$ but the other belonging to $\langle 10\bar{1}2\rangle$. The former is conventional contraction twinning for Mg, but the latter has an anti-twinning characteristic. Anti-twinning has been reported in BCC alloys, and its main difference from conventional twinning is the reversed twinning direction [3].

In addition, the sum of these two twin variants was $\langle 20\bar{2}0\rangle$ along the basal plane of the parent grain, which was also roughly parallel to the $\{10\bar{1}1\}$ lattice plane of the active twins, as outlined in the $\{10\bar{1}1\}$ pole figure (Supplementary Fig. 18). The calculated angle between the two twin boundaries adjoining the parent grain was 123.85°, which matched well with the TEM results. a-TIT should be composed of one parent grain and two conjugated contraction twins (Fig. 4j). Unlike i-TIT, these two twins are conventional; thus, the angle between the two TBs adjoining the parent grain is 56.15°, a supplementary angle with respect to the i-TIT type. The sum of these two twin variants was $\langle 000\bar{4}\rangle$ along the *c*-axis of the parent grain,

which was also roughly parallel to the $\{10\bar{1}3\}$ lattice plane of the active twins, as shown in the $\{10\bar{1}3\}$ pole figure (Supplementary Fig. 18).

Supplementary Note 4: Thermodynamic and kinetic reasons for the long-term stability of the TIT structure

The free energy of a supersaturated solid solution is higher than that of its equilibrium state. Therefore, dependent on a thermodynamic perspective, it has a tendency to decompose spontaneously to lower the system's free energy. However, although a supersaturated solid solution is thermodynamically unstable, an energy barrier must be overcome for it to transform into a more stable equilibrium state. At room temperature, this transformation process may be extremely slow, to the extent that it is virtually undetectable within the observation time. We will discuss this from the following aspects.

Firstly, from a thermodynamic perspective, the chemical free energy (G_{chem}) of a supersaturated solid solution is higher than that of the equilibrium state, resulting in a negative driving force for decomposition ($\Delta G_{\text{chem}} < 0$). Considering chemical free energy alone, the HCP structure is theoretically unstable and would spontaneously decompose towards equilibrium. However, the martensitic-like transformation does not produce isolated HCP structures but forms an interlocked network with triple twin junction nodes as the core. Consequently, the stability of this network is governed by the total Gibbs free energy (G_{total}):

$$G_{total} = G_{chem} + G_{interface} = G_{chem} + \sum_i \gamma_i A_i \quad (2)$$

where G_{chem} is the chemical free energy, $G_{\text{interface}}$ is the total interfacial energy, γ_{i} is the interfacial energy of the i-th type of interface, and A_{i} is the area of that interface. The interfaces between the HCP structure generated by martensitic-like transformation and the residual BCC parent phase are typically coherent or semi-coherent (with relatively low γ_{phase}). However, according to XRD refinement results, the volume fraction of β-Li in the HPHT-800 sample is very small, meaning A_{phase} is minimal. More importantly, the TIT structure contains a high density of $\{10\bar{1}1\}$ twins, whose boundaries are low-energy coherent interfaces in Mg alloys ($\gamma_{\text{twin}} \approx 84$ mJ/m^2). After martensitic-like transformation, the system enters a state composed of an extremely high density of low-energy interfaces (large A_{twin}, small γ_{twin}). For this structure to destabilize (e.g., through decomposition of the HCP structure or transformation into equilibrium phases), new phase interfaces with higher energy would have to be continuously created, thereby increasing $G_{\text{interface}}$. Furthermore, the growth and assembly

of β-Li particles would inevitably involve interface reconstruction, transforming the existing low-energy coherent TBs into high-energy large-angle β-Li GBs. This would cause a sharp increase in $G_{\mathrm{interface}}$, creating a substantial energy barrier that cannot be overcome at room temperature or even slightly elevated temperatures. This is supported by the TEM results that the fundamental configuration of the TIT structure remains intact, with no signs of detwinning, significant coarsening, or transformation observed after ageing at room temperature and slightly elevated temperatures (Supplementary Fig. 21).

Secondly, even if the system could overcome the aforementioned thermodynamic barrier and nucleate β-Li phase within the HCP supersaturated solid solution, its growth would require long-range diffusion of Li atoms through the HCP structure to reach the nucleation of β-Li. From a kinetic perspective, this diffusion process is governed by Fick's laws and thermal activation, with the diffusion coefficient D given by:

$$D = D_0 exp(-Q/k_B T) \tag{3}$$

where D_0 is the pre-exponential factor, Q is the activation energy for diffusion, k_B is the Boltzmann constant, and T is the absolute temperature. Crucially, the extensive TIT structure generated by martensitic transformation significantly complicates the diffusion paths for Li atoms, effectively increasing the effective activation energy (Q_{eff}). Additionally, the periodic arrangement of Li atoms along $\{10\bar{1}1\}$ TBs is clearly observable (Fig. 2d), indicating that a fraction of Li atoms are pinned at these boundaries after the martensitic-like transformation. Therefore, the diffusion coefficient D becomes exceedingly small due to a high Q_{eff} and the pinning effect at TBs at room temperature (low T),. This means Li atoms cannot achieve the compositional redistribution required for the decomposition of the HCP supersaturated solid solution.

Supplementary Note 5: Anisotropic strengthening of three structures

To elucidate the strengthening effect conferred by the three interface structures, the shear stress–strain behaviour of Mg–Li alloys featuring distinct interface configurations was investigated by MD with the LAMMPS package [4]. The MEAM potential was chosen to describe the interatomic interactions [5]. In this potential, the total energy of a system is given by:

$$E = \sum_i [F_i(\overline{\rho_i}) + \frac{1}{2}\sum_{j(\neq i)} S_{ij}\phi_{ij}(R_{ij}) \tag{4}$$

where F_{i} is the embedding function for atom i embedded in a background electron density $\overline{\rho_{\mathrm{i}}}$; and S_{ij} and $\phi_{\mathrm{ij}}(R_{\mathrm{ij}})$ are the screening function and the pair interaction between atoms i and j separated by a distance R_{ij}.

We combined the Atomsk code and our shell script to construct models of i-TIT, a-TIT, and general GBs. This model, with dimensions of $\phi60 \times 0.6$ nm^3, consists of three grains, which are connected to each other by i-TIT boundaries, a-TIT boundaries, or general GBs. Note that the Voronoi-Tesselation method has been performed to tuning the interface structure after contructuring the orientation relationship of three grains, ensuring its generality of GBs [5]. A $0.6 \times 80.0 \times 80.0$ nm^3 (x × y × z) supercell was employed to eliminate the internal stress that arose during artificial modelling. After relaxation for 60 ps using an isothermal–isobaric ensemble, the model was trimmed down to dimensions of $0.6 \times 45.0 \times 45.0$ nm^3. In particular, to explore the anisotropy of these interface strengthening effects, the relaxed structure was rotated by an increment of 60°, namely, 60°, 120°, 180°, 240°, and 300°, before trimming. The atoms within the outermost 0.5 nm layer along the y- and z-axes were anchored as fixed layers to avoid the influence of the extra interface introduced by the periodic boundary. The shear strain rate was 1.0×10^{-3} ps^{-1} with a maximum strain of 0.2. The canonical ensemble was employed to maintain a constant temperature. The purpose of maintaining a low temperature was to mitigate or eliminate the influence of thermal fluctuations on the atomic-scale deformation behaviour.

Supplementary Note 6: GB strengthening

The effect of the grain size on the yield stress (σ_{GB}) can be expressed as:

$$\sigma_{GB} = \sigma_0 + kd^{-\frac{1}{2}} \tag{5}$$

where σ_0 is the lattice friction stress, k is the Hall–Petch coefficient and d is the grain size. To calculate the yield strength of the HPHT-800 Mg-9Li alloy with a grain size of ~100 μm, the values of σ_0 and k were taken from the Mg matrix, for which σ_0 is approximately 20.6 MPa and k is 300 MPa $\mu m^{1/2}$. Therefore, σ_{GB} was calculated to be 50.6 MPa. This value was similar to the yield strength of the as-cast Mg-9Li alloy, indicating that the grain size was not significantly affected by the HPHT process. Note that the contribution of grain boundary strengthening was far lower than the actual measured value (500 MPa). Therefore, we focused on the contribution of the triple nanotwins to strengthening the HPHT-800 Mg-9Li alloy.

Supplementary Note 7: TIT strengthening

Twin strengthening is essentially determined by the dislocation–twin interaction mechanism. Specifically, dislocations can be pinned by TBs or transmitted across them. The nonuniform partial dislocation extension (NPDE) model [6] and confined layer slip (CLS) model [7] can be used to estimate the triple nanotwin strengthening effect. The strengthening effect of the triple nanotwins on the yield stress (σ_{TT}) can be expressed as:

$$\sigma_{TT} = \sigma_{NPDE} + \sigma_{CLS} \tag{6}$$

$$\sigma_{NPDE} = \frac{E}{b} + \beta \frac{Gb}{\lambda} \tag{7}$$

$$\sigma_{CLS} = \frac{Gb[1-v\cos^2(\varphi)]\sin\phi}{2\pi\lambda(1-v)} \ln\frac{\alpha\lambda}{b} \tag{8}$$

where σ_{NPDE} and σ_{CLS} are the contributions of the NPDE and CLS models, respectively; E is the stacking fault energy of the Mg matrix; G is the shear modulus; **b** is the magnitude of the Burgers vector; β is a material constant; λ is the twin thickness; v is Poisson's ratio; φ is the misorientation between the dislocation line and the Burger vector; ϕ is the angle between the slip plane and the twin plane; and α is the dislocation core parameter. These values are listed in Supplementary Table 6. On the basis of equations (6-8), the total contributions of the grain boundary and TIT strengthening were 501.6 MPa, where the contributions of GBs and TIT were approximately 50.6 MPa and 451 MPa, respectively.

Supplementary Table 1. Actual chemical compositions (wt. %) of as-cast and HPHT-800 Mg-9Li alloys by ICP-MS.

Alloy (wt.%)	Mg	Li	Fe	Ni	Cu	Bal.
As-cast	91.11	8.87	0.005	0.001	0.002	0.012
HPHT-800	91.34	8.63	0.005	0.002	0.003	0.020

Supplementary Table 2 | Disclination solutions for twin boundary junctions formed by C1/C2/C3.

Type	C_2/C_1	C_2/C_3	C_3/C_1	Disclination
1	$(1\bar{1}0\bar{1})_{C1}$	$(\bar{1}10\bar{1})_{C3} \parallel (1\bar{1}0\bar{1})_{C2} \leftrightarrow (000\bar{1})_{C1}$	$(1\bar{1}01)_{C1}$	11.6°
2	$(1\bar{1}0\bar{1})_{C1}$	$(\bar{1}103)_{C3} \parallel (\bar{1}10\bar{3})_{C2} \leftrightarrow (\bar{1}100)_{C1}$	$(1\bar{1}01)_{C1}$	3.7°

Supplementary Table 3 | Yield strength of Mg-Li based alloys prepared by different processing techniques.

Composition (wt.%)	State	YS (MPa)	Elongation (%)	Ref.
Mg-14Li-6Zn-2Y	As-cast	105.3	19.4	8
Mg-14Li-6Zn-2Gd	As-cast	116.5	26.1	
Mg-7Li-5Y	As-cast	150	5.5	9
Mg-8Li-3Al-2Zn-0.5Y	As-cast	165.1	13.9	10
Mg-8Li-3Al-2Zn-0.5Y	Rolled	230	7.1	10
		205.3	8.2	
Mg-7Li-2Al-0.1Y	Rolled	250	15.7	11
Mg-9Li-5Al	Extruded	185	33.2	12
Mg-9Li-5Al-0.4Sm	Extruded	220	16	
Mg-5Li-5Al-2Zn-0.5Y	Extruded	186.4	16.1	13
Mg-4Li-3Al-2Zn	SPDed	350	6	14
		320	7	
Mg-10Li-3Al-2Zn	SPDed	168.2	22.4	15
		171.8	26.7	
		204.3	22.56	
Mg-11Li-3Al	Aged	360	6	16
Mg-11Li-3Al-0.2Zr-0.6Y	Aged	240	6.5	17
	Aged+rolled	224	25.7	
Mg-14Li-7Al-1Y	Aged	453	3.8	18
Mg-13Li	HPHTed	305	21	19
Mg-8Li	HPHTed	249	23.6	20
Mg-9Li	HPHTed	443~508	6.2~7.5	This work

Supplementary Table 4 | Specific yield strength (SYS) of metal materials prepared by different processing techniques.

Composition (wt.%)	State	SYS (kNmkg^{-1})	Elongation (%)	Ref.
Al-0.9Mg-1Si-0.6Cu-0.7Mn	Aged 1h	101.8	18	21
	Aged 2h	109.1	11.8	
	Aged 3h	130.9	8	
	Aged 6h	145.5	5.6	
	Aged 9h	138.2	9	
Al-1.1Mg-0.9Si-0.5Cu-0.2Zn	Rolled + aged	126	7.1	22
Al-1.1Mg-0.9Si-0.5Cu -0.2Zn-0.5Mn		137.8	16	
Al-1.1Mg-0.9Si-0.5Cu-0.2Zn -0.5Mn-0.2Cr		137.1	18.5	
Al-1.1Mg-0.9Si-0.5Cu-0.2Zn -0.5Mn-0.2Ni		135.1	10.7	
Ti-6Al-4V	3D print	220.4	5.1	23
	3D print +ultrasound	250	4.8	
Ti-5Al-5Mo-4V-1Cr-1Fe	Additively manufactured	241	7.5	24
		220	8	
		180	18	
		200	11	
Fe-15Cr-3Mn-3Ni	Additively manufactured	163.2	4	25
Fe-3Cr-1Ni-1Si-1Mo-0.7Mn	Additively manufactured	152	7.7	26
Fe-14Co-11Ni-3Cr-1Mo	Heat treatment	219.5	11	27
Fe-5Cr-1Mo-1Si-1V-0.5Mn	Additively manufactured	202.5	4.2	28
Mg-10Gd-1.7Y-1Zn-0.5Zr	Solid solution	170.5	13.6	29
	Peak aged	158	9	
	Aged	135.6	16.2	
Mg-14Li-7Al-1Y	Peak aged	350	5	18
Mg-5.4Sn-4.2Zn-2Al -0.1Na	Peak aged	142	4	30
Mg-5.4Sn-4.2Zn-2Al -0.2Mn-0.1Na	Peak aged	187.56	3	
	Extruded	134.4	10	
Mg-5.4Sn-6Zn-2Al	Single aged	137.8	20	
	Double aged	190	15	

Mg-6.6Sn-6Zn-2Al<br>-0.2Mn	Single aged	147.3	13	
	Double aged	200	14	
Mg-13Li	HPHTed	232.3	22	[19]
Mg-8Li	HPHTed	173.6	24	[20]
Mg-9Li	HPHTed	350	6.2~7.5	This work

Supplementary Table 5 | Shear strength values in various directions.

Angle (°)	Shear strength (GPa)		
	i-TIT	a-TIT	GB
0	1.191	1.081	0.865
60	1.212	1.149	1.128
120	1.235	1.265	1.245
180	1.191	1.074	0.856
240	1.221	1.163	1.107
300	1.194	1.236	1.229

Supplementary Table 6 | Parameters used to evaluate the yield stress contributed by triple nanotwins.

E (mJ/m^2)	G (GPa)	b (nm)	β	λ (nm)	v	φ (°)	ϕ (°)	α
100	16.6	0.32	0.3	50	0.35	60	56.3	$\sqrt{2}$

References

1 Wei, Y. *et al.* A systematic study of disclination-induced internal stress associated with multiple types of twin-twin reactions in Mg alloys: Analytical solutions and numerical calculations. *Acta Mater.* **284**, 120619 (2025).

2 Hielscher, R. & Schaeben, H. A novel pole figure inversion method: specification of the MTEX algorithm. *J. Appl. Cryst.* . **41**, 1024-1037 (2010).

3 Wang, J. *et al.* Anti-twinning in nanoscale tungsten. *Sci. Adv.* **6**, eaay2792 (2020).

4 Plimpton, S. Fast parallel algorithms for short-range molecular dynamics. *J. Comput. Phys.* **117**, 1-19 (1995).

5 Kim, Y. M., Jung, I. H. & Lee, B. J. Atomistic modeling of pure Li and Mg-Li system. *Model. Simul. Mater. Sci. Eng.* **20**, 035005 (2012).

6 Gu, P., Dao, M., Asaro, R. J. & Suresh, S. A unified mechanistic model for size-dependent deformation in nanocrystalline and nanotwinned metals. *Acta Mater.* **59**, 6861-6868 (2011).

7 Ying, P. *et al.* Enhancing the hardness of diamond through twin refinement and interlocked twins. *Nat. Synthesis.* **4**, 391-398 (2025).

8 Liu, W. *et al.* Microstructural Evolution and Mechanical Properties of As-Cast and As-Extruded Mg-14Li Alloy with Different Zn/Y and Zn/Gd Addition. *Adv. Eng. Mater.* **22**, 2000480 (2020).

9 Dong, H., Wang, L., Wu, Y. & Wang, L. Effect of Y on microstructure and mechanical properties of duplex Mg-7Li alloys. *J. Alloy. Compd.* **506**, 468-474 (2010).

10 Liu, W. *et al.* Effect of rolling strain on microstructure and tensile properties of dual-phase Mg-8Li-3Al-2Zn-0.5Y alloy. *J. Mater. Sci. Technol.* **34**, 2256-2262 (2018).

11 Cao, F., Zhou, B., Ding, X., Zhang, J. & Xu, G. Mechanical properties and microstructural evolution in a superlight Mg-7.28Li-2.19Al-0.091Y alloy fabricated by rolling. *J. Alloys Compd.* **745**, 436-445 (2018).

12 Hu, Z. *et al.* Influence of Sm addition on microstructural and mechanical properties of as-extruded Mg-9Li-5Al alloy. *J. Alloys Compd.* **842**, 155836 (2020).

13 Tang, Y., Le, Q., Misra, R. D. K., Su, G. & Cui, J. Influence of extruding temperature and heat treatment process on microstructure and mechanical properties of three

structures containing Mg-Li alloy bars. *Mater. Sci. Eng., A.* **712**, 266-280 (2018).

14 Yang, Y. *et al.* Achieving ultra-strong Magnesium–lithium alloys by low-strain rotary swaging. *Marer. Res. Lett.* **9**, 255-262 (2021).

15 Li, Y. *et al.* Effect of processing parameters on the microstructure and tensile properties of a dual-phase Mg-Li alloy during friction stir processing. *J. Mater. Res. Technol.* **17**, 2714-2724 (2022).

16 Tang, S. *et al.* Precipitation strengthening in an ultralight magnesium alloy. *Nat. Commun.* **10**, 1003 (2019).

17 Xu, W. *et al.* A high-specific-strength and corrosion-resistant magnesium alloy. *Nat. Mater.* **14**, 1229-1235 (2015).

18 Xin, T. *et al.* Ultrahigh specific strength in a magnesium alloy strengthened by spinodal decomposition. *Sci. Adv.* **7**, eabf3039 (2021).

19 Peng, Q. *et al.* Interactive contraction nanotwins-stacking faults strengthening mechanism of Mg alloys. *Acta Mater.* **169**, 36-44 (2019).

20 Fu, H. *et al.* Achieving High Strength and Ductility in Magnesium Alloys via Densely Hierarchical Double Contraction Nanotwins. *Nano Lett.* **17**, 6117-6124 (2017).

21 Li, Y. *et al.* Effect of mechanical properties, microstructure and residual stress on the bending springback behavior of high-strength Al-Mg-Si-Cu alloy tubes. *J. Mater. Res. Technol.* **28**, 3609-3618 (2024).

22 Ding, L. *et al.* Influence of the combined additions Mn, Cr or Ni on the formation of dispersoids and mechanical properties of Al-Mg-Si-Cu alloys. *Mater. Sci. Eng., A.* **892**, 145962 (2024).

23 Todaro, C. J. *et al.* Grain structure control during metal 3D printing by high-intensity ultrasound. *Nat. Commun.* **11**, 142 (2020).

24 Li, D. *et al.* Additive manufacturing of high strength near β titanium alloy Ti-55511 by engineering nanoscale secondary α laths via in-situ heat treatment. *Mater. Sci. Eng., A.* **814**, 141245 (2021).

25 Lehnert, R. *et al.* Microstructural and mechanical characterization of high-alloy quenching and partitioning TRIP steel manufactured by electron beam melting. *Mater. Sci. Eng., A.* **794**, 139684 (2020).

26 Seede, R. *et al.* An ultra-high strength martensitic steel fabricated using selective laser melting additive manufacturing: Densification, microstructure, and mechanical properties. *Acta Mater.* **186**, 199-214 (2020).
27 Ran, X.-z. *et al.* Effects of post homogeneity heat treatment processes on microstructure evolution behavior and tensile mechanical properties of laser additive manufactured ultrahigh-strength AerMet100 steel. *Mater. Sci. Eng., A.* **723**, 8-21 (2018).
28 Rafi, H. K., Pal, D., Patil, N., Starr, T. L. & Stucker, B. E. Microstructure and Mechanical Behavior of 17-4 Precipitation Hardenable Steel Processed by Selective Laser Melting. *J. Mater. Eng. Perform.* **23**, 4421-4428 (2014).
29 Wang, H. *et al.* An ultra-high strength and toughness as-cast Mg-10Gd-1.7Y-1Zn-0.5Zr alloy. *J. Magnes. Alloys.* **13**, 894-901 (2025).
30 Sasaki, T. T. *et al.* Strong and ductile heat-treatable Mg-Sn-Zn-Al wrought alloys. *Acta Mater.* **99**, 176-186 (2015).
