# Supplementary material for: Triple-interlocked-nanotwinned bulk magnesium alloys with exceptional strength and ageing resistance
Source: Nat Commun. 2026 May 22;17:4775. doi: 10.1038/s41467-026-73640-w (PMC13219722; doi:10.1038/s41467-026-73640-w)
Supplement: Supplementary file 2 — Description of Additional Supplementary Files [file 41467_2026_73640_MOESM2_ESM.pdf]

Description of Additional Supplementary Files

Supplementary Movie 1: 3D tomography of TIT structure. The incident beam was successively tilted onto the $\{10\bar{1}0\}$, $\{10\bar{1}1\}$, $\{10\bar{1}2\}$, $\{11\bar{2}0\}$, $\{10\bar{1}3\}$, $\{20\bar{2}0\}$, $\{20\bar{2}1\}$, $\{0004\}$, and $\{20\bar{2}2\}$ Mg diffraction rings; for every ring the beam was azimuthally swept through 0-360° in 2° increments. The grains were segmented using a 5° misorientation threshold.

Supplementary Movie 2: TIT formation mechanism. The formation process of nanotwins induced by phase transformation in Mg-9Li alloy with a tensile strain rate of 1.0×10^{-3} ps^{-1} and the maximum strain reached 0.25. In the video, blue represents the α-Mg matrix of HCP structure, red represents the β-Li of BCC structure, and yellow represents defects such as FCC structures or stacking faults. Moreover, twins formed in the video were $\{10\bar{1}1\}$ contraction twins.

Supplementary Movie 3: TIT deformation mechanisms. Three structures, relative to i-TIT, a-TIT and GBs. A shear deformation was performed along the y-axis. The shear strain rate was 1.0×10^{-3} ps^{-1} with the maximum strain reaching 0.2. In videos, interface structures and defects are represented by gray and yellow atoms respectively.

Supplementary Movie 4: In situ compression process of as-cast and HPHT-800 Mg-9Li alloy nanopillars with diameter of 300 nm at 300 kV. A cubed-corner diamond indenter with a 1 μm tip was used for all measurements performed at load-rate control with a peak force of 1 mN and a strain rate of 8.3×10^{-3} s^{-1}.
